# A novel computational biostatistics approach implies impaired dephosphorylation of growth factor receptors as associated with severity of autism

**DOI:** 10.1038/tp.2013.124

**Published:** 2014-01-28

**Authors:** K M Wittkowski, V Sonakya, B Bigio, M K Tonn, F Shic, M Ascano, C Nasca, G Gold-Von Simson

**Affiliations:** 1 The Rockefeller University, Center for Clinical and Translational Science, New York, NY, USA; 2 Hochschule Koblenz, RheinAhrCampus, Joseph-Rovan-Allee 2, Remagen, Germany; 3 Yale School of Medicine, Yale Autism Program, New Haven, CT, USA; 4 Tuschl Laboratory of RNA Molecular Biology, The Rockefeller University, New York, NY, USA; 5 McEwen Laboratory of Neuroendocrinology, The Rockefeller University, New York, NY, USA; 6 New York University, Langone Medical Center, New York, NY, USA

**Keywords:** genetics, autism, epilepsy, computational biostatistics, genome-wide significance, genome-wide association studies, minor allele frequency

## Abstract

The prevalence of autism spectrum disorders (ASDs) has increased 20-fold over the past 50 years to >1% of US children. Although twin studies attest to a high degree of heritability, the genetic risk factors are still poorly understood. We analyzed data from two independent populations using *u*-statistics for genetically structured wide-locus data and added data from unrelated controls to explore epistasis. To account for systematic, but disease-unrelated differences in (non-randomized) genome-wide association studies (GWAS), a correlation between P-values and minor allele frequency with low granularity data and for conducting multiple tests in overlapping genetic regions, we present a novel study-specific criterion for ‘genome-wide significance'. From recent results in a comorbid disease, childhood absence epilepsy, we had hypothesized that axonal guidance and calcium signaling are involved in autism as well. Enrichment of the results in both studies with related genes confirms this hypothesis. Additional ASD-specific variations identified in this study suggest protracted growth factor signaling as causing more severe forms of ASD. Another cluster of related genes suggests chloride and potassium ion channels as additional ASD-specific drug targets. The involvement of growth factors suggests the time of accelerated neuronal growth and pruning at 9–24 months of age as the period during which treatment with ion channel modulators would be most effective in preventing progression to more severe forms of autism. By extension, the same computational biostatistics approach could yield profound insights into the etiology of many common diseases from the genetic data collected over the last decade.

## Introduction

Autism spectrum disorders (ASDs) include a broad range of developmental brain disorders that share a complex and heterogeneous etiology characterized by fundamental deficits in social reciprocity, impaired language and communication skills, as well as repetitive and stereotypic behavior. About 1% of the population are directly affected and many more as family members. Despite the high heritability of ASD, with near-perfect concordance in monozygotic twins,^1^ a >50% heritability among siblings^2^ and a >25% risk for developing ASD in a male sibling,^3^ the genetic risk factors are still poorly understood.^4^ In the absence of reliable and feasible biomarkers, ASDs are still diagnosed exclusively according to behavioral criteria. ASD-specific therapeutic approaches are urgently needed to meet the challenge of an increasing prevalence, yet genome-wide association studies (GWAS) have not met the need for a better understanding of the etiology of ASD.^5^

Many GWAS have been marred by both low sensitivity and specificity. The first three studies reported *SYT17* (*s*=−log_10__P_*P*=6.72), *DMD* (6.57),^6^ the moesin pseudogene *MSNP1AS* (9.67)^7^ (>750  kB from either of the cadherins *CDH9* or *CDH10*),^6^ an independent finding in *MSNP1AS* (5.47),^8^ and the taste receptor *TAS2R1* (6.68).^9^ The ‘largest (GWAS) of psychiatric illness so far in 33 332 subjects with psychiatric disorders, including 4949 subjects with ASD,^10^ pointed to two calcium (Ca^2+^) channel subunits (*CACNA1C* and *CACNB2*, five disorder meta-analysis only), consistent with previously suggested involvement of Ca^2+^ signaling in psychiatric disorders, but also to an intron in *AS3MT*, a gene involved in arsenic metabolism. The models with the best fit to ASD suggested *TCF4* (a transcription factor, which ‘may have an important role in nervous system development'), but also *DPYD* (a pyrimidine catabolic enzyme) and *PCGEM1* (non-protein coding). In an even larger study of educational attainment in 126 558 subjects, only ‘three independent SNPs were genome-wide significant'.^11^

Our results are based on one of the largest studies of ASD in the United States, which included 2705 children with ASD from the Autism Genome Project (AGP).^12^ In the original analysis, both stages confirmed *CNTNAP2* as a risk factor for ASD,^13^ but the ‘highly associated *MACROD2* SNP from primary stage 1 analysis showed little if any signal in the stage 2 sample (*P*-value 0.206)'.^12^ Even a score combining several putative risk alleles could not account for more than 1% of the variance.^12^ Another analysis of the first stage (AGP I)^14^ found seven rare *de novo* copy-number variations (CNV) unique to cases, four among them in a cluster of genes related to neurexin/neuregulin signaling comprising *SHANK2* (two cases), a region containing five genes, including *SYNGAP1* (one) and *DLGAP2* (one), as well as a group of 7 among 219 rare inherited CNVs (*P*=3.1 × 10^−^^3^) in a 300 -kB X-linked region containing *PTCHD1*, a gene with unknown function deleted in two related boys with intellectual disability.^15^ Overall, CNVs were enriched in genes ‘involved in cellular proliferation, projection and motility, and GTPase/Ras signalling', yet no ‘connected pathways' could be postulated.^14^

Despite evidence for a likely involvement of *de novo* and environmental or epigenetic risk factors, including maternal antibodies^16^ or stress during pregnancy^17^ and paternal age,^18,19^ we contend that coding variations contribute substantially to the heritability of ASD and can be successfully detected and assembled into connected pathways with GWAS—if the experimental design, the primary outcome, the statistical methods used, and the decision rules applied were better targeted toward the particulars of non-randomized studies of common diseases. With *u*-statistics for genetically structured wide-locus data comprising several neighboring SNPs (μGWAS) addressing the former two conditions, we have recently confirmed axonal guidance and Ca^2+^ signaling as key pathways in childhood absence epilepsy (CAE)^20^ from 185 cases and publicly available controls only. As shared genetic risk factors have been suggested for neurodevelopmental disorders, in general,^21^ and epilepsies and ASD, in particular,^22, 23, 24^ we hypothesized that these pathways are involved in ASD as well. In this study, we took advantage of the higher power of μGWAS as a wide-locus approach and its higher specificity as a non-parametric method, and compared more vs less severe cases, to elucidate risk factors for particular features of ASD. Finally, we present a novel objective decision rule for study-specific genome-wide significance, which adjusts for a GWAS-specific bias in determining cutoffs for enrichment with disease-related genes.

## Materials and methods

### Study subjects/genotyping

The study was approved by the IRB of The Rockefeller University. No human participants were involved in the research. The samples were genotyped on Human1Mv1_C and Human1M-Duov3_B Illumina chips. The genomic data were downloaded from dbGaP (data set phs000267.v2.p2) and details of the study population are described elsewhere.^12,25^ In the preparation of the data, we retained only SNPs with an rsID. During quality control, we removed SNPs according to the following criteria (AGP I/AGP II): MAF<2% (851/1066), >20% missing genotypes (5179/791), one-sided Hardy–Weinberg *P*-values for lack of heterozygosity <1 × 10^−^^4^ (0/0), LD with their neighbors >0.98 (149  512/149  982), data quality μ-score^20^ among the bottom 10% (67 979/68 380).

### Study design

We aimed at risk factors specific to strict definition autism (SDA) by comparing case subpopulations meeting the definition of SDA and milder cases with ASD (excluding SDA), for which we here use the term ‘high-functioning autism' (HFA). To reduce variance, we included only subjects of European ancestry genotyped on the more frequently used platform in either stage. In AGP II, we also excluded female cases because of confounding between chip platform and disease severity. The total number of subjects included (m:male/f: female) was 547/98 (SDA) and 358/68 (HFA) in AGP I and 375 (SDA) and 201 (HFA) in AGP II.

### Wide-locus approach

To overcome several of the shortcomings seen in previous applications of single-SNP GWAS (ssGWAS) to common diseases, we combined several strategies at different stages of the analysis process. We aimed at wide loci of up to six neighboring SNPs as a primary outcome and applied the same non-parametric GWAS approach based on *u*-statistics for multivariate data^26^ with genotypic structures (μGWAS)^20^ as in the previous CAE study.^20^ For the AGP I data, we stratified the analysis by sex,^27^ and selected sex-specific results, if either sex, after Bonferroni correction for two sexes,^28^ was more significant than the stratified analysis. To avoid spurious findings, we excluded loci outside of linkage-disequilibrium (LD) blocks containing genes with known function or adjacent to their 5′-end and also loci highly influenced by a single SNP only, unless these SNPs were implicated in both stages or had been implicated in other studies.

### MAF significance correlation

With any finite sample size, the significance of a *u*-/rank test is limited and more significant results can only be obtained for SNPs with sufficiently high MAF. We performed ssGWAS simulations with 2 500 000 permuted phenotypes, comparing two groups of equal size *n* for various MAFs. The 1–10^−5^ quantile of the permutation distribution drops from the expected *s*=5.26 cutoff, which is routinely met for MAF >0.33, to 4.9 (*n*=1000 subjects), 4.7 (*n*=500) and 4.5 (*n*=300) for a MAF of 0.05. For the 7.5 level, this bias is projected to be even larger. Because of this MAF significance correlation, the expected diagonal in a ssGWAS quantile-quantile (QQ) plot under the null hypothesis that ‘*no SNP is associated with the trait*',^29^ turns into a curve dropping below the diagonal towards the end.

Estimating the expected *s*-value distribution from >10^8^ permutations to obtain stable estimates of the 1–10^−7.5^ quantile is neither practical nor sufficient to avoid a biased selection of SNPs for limited tests. Because of the MAF significance correlation, any SNP that is ‘significant' when comparing observed phenotypes, is also more likely to be ‘significant' with random phenotype permutations (see Supplementary Figure 12).

### Non-randomization bias

The reason for this curvature often not being recognized in QQ plots is that GWAS subjects are deterministically categorized based on their outcome (here: SDA vs HFA), rather than randomly assigned to interventions (as in clinical trials). Any deterministically categorized populations, however, are expected to differ systematically in aspects related to neither the condition of interest nor common ancestry factors (which could potentially be accounted for through principal component analysis). When the downward trend from using a limited test and the upward bias from deterministic selection are similar, the *s*-values may still appear to follow the diagonal, hiding loci suggesting ‘true association'.^29^

### Multipicity adjustments for diplotype length

**Figure 1:**
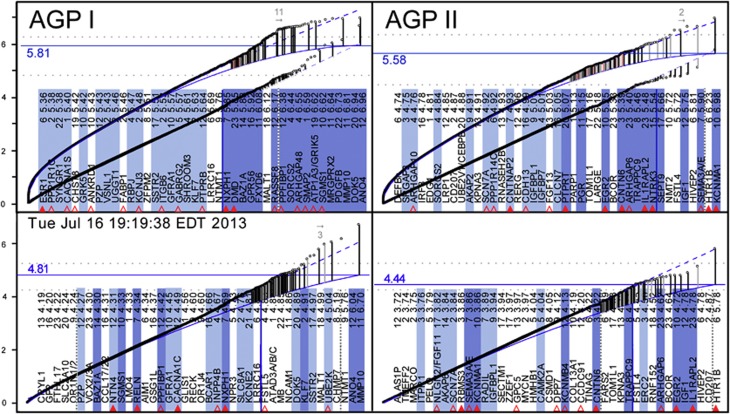
μGWAS QR plot (curved) vs traditional ssGWAS QQ plot (straight), left: AGP I, right: AGP II. Each point represents the most significant result among all diplotypes centered at the same SNP ranked by significance (low to high). Dashed blue curve: projection. Solid blue curve: loess estimation (see Materials and Methods). Vertical lines connect the most significant *s*-values (−log_10_ P) of a gene (dot) with its expected value (solid blue line). Light red and gray vertical lines indicate genes with unknown function and results with low reliability (either low μIC or reliance on a single SNP), respectively. Top and bottom gene list (by significance, right to left, excluding genes with unknown function): μGWAS and ssGWAS results, respectively. Shaded genes are among the genes highlighted in Figure 2. Full and open triangles mark genes with an identical match or family member of *SFARI* genes, respectively (see Supplementary Table 1 for details). The dotted horizontal lines represent the projected WG apex (6.272 and 6.064) and an exploratory 100 gene cutoff (4.835 and 4.480) for AGP I and AGP II, respectively (Supplementary Table 1). The horizontal solid blue line indicates the proposed study-specific GWS.

For multivariate tests of overlapping diplotypes, the estimated quantile-rank (QR) curve needs to be elevated above the diagonal throughout to account for multiple tests conducted around the same SNP. Because most of these tests are highly dependent, the elevation of the estimated QR curve compared with the estimated QQ curve (Figure 1) is limited, but the distance is likely to vary across diseases and populations.

### Projected QR curves

The diagonal of the traditional QQ plot does not depend on any data, including the most ‘significant' data. The *s*-values are expected to fit the diagonal for the most part (except for the most significant results),^29^ (Figure 1A), as the vast majority of SNPs are expected not to be associated with the disease. In direct analogy, the QR curve for a multivariate test should be ‘smooth', with upward deviations indicating ‘true association', related or not. On the basis of the above rationale and the simulation results shown below, we propose to estimate the highest point of the projected QR curve (apex) for each chromosome from a smooth projection of the *s*-values after truncating as many of the highest values as needed for the projection to have a monotone increase and, conservatively for a limited test, a non-positive second derivative. Fitting against the data also reduces the effect of population stratification^29^ (Figure 1B). (For computational convenience, we have chosen locally weighted polynomial regression,^30^ as implemented in S+, loess.smooth (…, degree=2, family='gaussian').)

### Estimated WG QR apex

While chromosomes may differ with respect to their content of related and unrelated risk factors (take for example, the HLA region in autoimmune diseases), random errors are expected to have the same distribution across all autosomes. Hence, we can estimate the expected WG apex as the (winsorized) median projected apex among autosomes with the smallest deviation of *s*-values from the projection. (Here, we selected ten autosomes based on maximum norm and median for robustness, but the strategy to determine the optimal number, including the criteria for ‘optimality', remains to be determined.)

### Estimated QR curves

The estimated curve for each chromosome is then calculated as the loess projection^30^ of this chromosome's *s*-values with as many of the highest values replaced with the estimated WG apex until the curve's apex is at or below that level. Applied to the WG projection (Figure 1, Supplementary Figures 5-11, bottom right), this procedure yields the estimated WG curve. The simulation results in Supplementary Figure 12 (bottom right vs bottom left) demonstrate the low variance of the estimates from phenotype permutations and the similarity of their median apex with the winsorized median apex estimated from the observed *s*-values. Details of this approach are discussed in the Supplementary Material in the context of Supplementary Figure 5.

### Study-specific GWS

For studies aiming to confirm individual SNPs as associated with a phenotype, the ‘confirmatory' paradigm^31^ requires adjustment for multiplicity. When applied to GWAS, these adjustments are typically based on a ‘customary' fixed 0.05 level, irrespective of study size or relative risk of type I over type II errors (see, Fisher^32^ p. 358 and Gigerenzer^33^ for a discussion), and the assumption of 1 000 000 independent SNPs, irrespective of chip density.^29^ Moving from individual SNPs to overlapping diplotypes increases the dependency of any formal multiplicity adjustment on assumptions with questionable biological validity.

In most GWAS, however, we do not aim to confirm hypotheses regarding specific SNPs. Instead, we aim at picking likely candidates from >40 000 (pseudo-) genes, whose relative importance and epistatic interactions are unknown. As graphical procedures are particularly useful for such ‘exploratory' studies,^34^ we chose QR plots to guide with interpretation. Unfortunately, exact cutoffs for deviation of *s*-values from the estimated curve are unknown. When ‘the knowledge [is] at best approximate[,] an approximate answer to the right question, which is often vague, [is far better] than an exact answer to the wrong question, which can always be made precise',^35^ pp 13–14). Hence, we present a heuristic approach that relies on fewer unrealistic assumptions than typical attempts to quantify a particular error rate.

As the expected WG curve needs to be estimated, the *s*-values have a complex dependency structure, and the appropriate level of significance (α) for the given sample size is unknown, we propose a heuristic decision rule based on weak assumptions only. In the long run, one would expect most *s*-values above the apex to be significant at any α > 0 (consistency) and regions with the strongest association to have the highest odds at being included (unbiasedness). Hence, for a particular α, one could lower the cutoff, but, to account for variance in estimating the apex, one would need to raise it. As a compromise, we propose the estimated WG apex as a cutoff for study-specific GWS.

## Results

### Traditional GWS cutoffs

The commonly used cutoffs tend to have low sensitivity for enriched genes. In an analysis of AGRE/NIMH data,^9^ for instance, 'excess of independent regions associated at *P*<10^−^^5^', had been observed, even though ‘no SNP met criteria for genome-wide [permutation] significance [of] *P*<2.5 × 10^−7^'. None of our results exceeds this cutoff, either, even though our ssGWAS results (Figure 1, bottom) and, in particular, μGWAS results (Figure 1, top list) of both stages are also highly enriched with genes collected in the SFARI Gene database (Figure 1, red triangles).

The WG projection apices for ssGWAS of ~6.0 (Figure 1, bottom) are clearly exceeded only once, by *MMP10* in AGP I. A noticeable deviation from the expected distribution is commonly used as a decision criterion for selecting candidate genes.^29^ On the basis of the projected WG curve, only three AGP I genes (Table 1a), but none of the AGP II genes, fulfill this criterion in ssGWAS, compared with eleven and two genes (Table 1a/b, excluding *NTMT1*), respectively (Figure 1, top), in μGWAS.^20^

### Study-specific GWS

The proposed more flexible cut-offs (see Materials and Methods) account for the MAF significance correlation. In both AGP stages, ~100 genes deviate from the estimated curve (Figure 1, solid curve) as an heuristic criterion for expected enrichment^37^ (Supplementary Table 1). The set of genes deviating sufficiently, however, can be difficult to determine objectively. We are proposing the estimated WG QR apex as a more formal study-specific criterion, which here increases the number of significant regions from none (when compared against a fixed GWS of 7.5) to 18 and 8 for AGP I and II, respectively (Figure 1, solid horizontal lines).

In all μGWAS included, that is, Figure 1 (four analyses), Figure 4, Supplementary Figures 7,9 and 12 (three analyses), as well as in numerous others (results not shown), <20 genes or gene regions exceeded study-specific GWS. The high enrichment with pathway genes even below the WG apex in μGWAS of AGP II (Figure 1, top right) attests to the proposed approach being conservative. Further support comes from the number of selected genes being smaller with randomized vs observed phenotypes (3–7 vs 14, Supplementary Figure 12), albeit not zero, consistent with the above MAF significance correlation resulting in SNPs with a high MAF not only to be more likely to be significant with observed phenotypes, but also with random phenotype permutations. In addition, the number of selected genes is smaller with comparable populations of smaller size (Figure 1, Figure 4 vs Figure 1), as expected in selection procedures.^38,39^

The previous CAE study and the additional comparison of HFA cases vs parental controls (Figure 4) had a study-specific cutoff of 7.20 (Supplementary Figure 7, 21 functional regions, including *CNTNAP2*, *DLGAP1* and *NALCN* as 19th to 21st), and 4.91 (Supplementary Figure 8, 25 regions, including *ARHGAP24*, *SLC25A21* and *PTENP1* as 25th, 22nd and 20th), respectively.

### Specificity of the proposed approach in the current study

**Figure 2:**
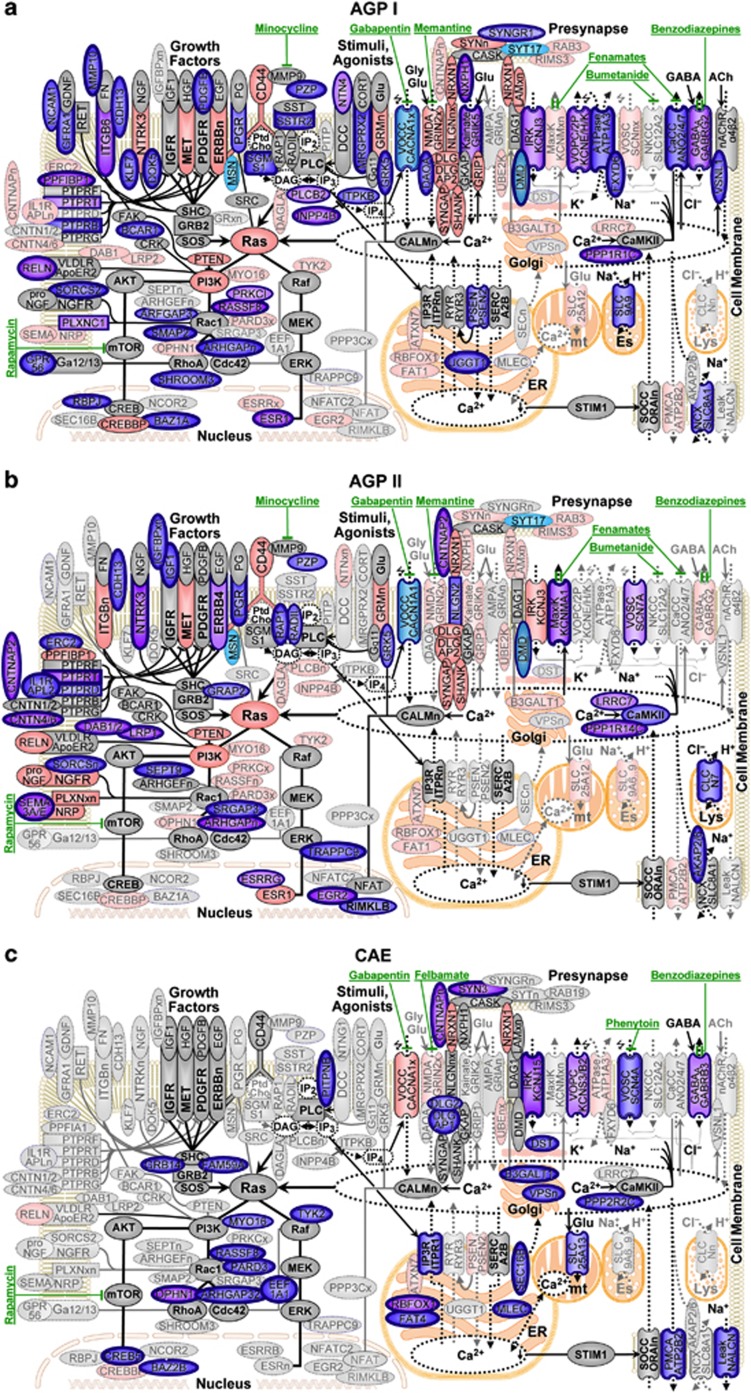
Ras/Ca^2+^ signaling in ASD and CAE. (**a**) AGP I, (**b**) AGP II, (**c**) CAE. Pathway-related genes among the top 20, 50 and 100 are circled in bold, double and thin blue lines, respectively. Genes included in SFARI Gene (ASD) and CarpeDB (CAE), respectively, are shaded in red (see Supplementary Table 1 for details); the five genes identified in previous GWAS (see Introduction) are indicated in turquoise and underlined. Upon GF binding to cell-surface receptors (for example, *IGFR*, *MET*, *PDGFR*, *ERBBn*), formation of receptor complexes initiates proliferation, cytoskeletal organization and survival along Ras downstream effectors. GFs are immediately deactivated by PTPRs. Downstream activities are modulated by agonists binding to G-protein-coupled receptors (GPCR) activating phospholipase C (*PLC*) to form membrane diacylglycerol (DAG) and inositol trisphosphate (IP_3_). While DAG activates Ras directly, IP_3_ stimulates (‘winged' arrows) the release of Ca^2+^ from the endoplasmic reticulum (ER), starting a process of Ca^2+^-dependent activation of Ras involving several feedback loops. The fall of Ca^2+^ concentration in internal stores (dotted areas) leads, via *STIM1*, to the opening of store-operated Ca^2+^ channels (SOCC) in the plasma membrane. *ITPKB* phosphorylates IP_3_ into IP_4_, which opens voltage-operated Ca^2+^ channels (VOCC). CaCCs can either directly activated by Ca^2+^ elevation or through Ca^2+^/calmodulin kinase II (CaMKII)-mediated phosphorylation. Other plasma membrane ion channels involved are Ca^2+^ channels operated by NMDA and kainate ligands, voltage-operated potassium channels (VOPC). GABA-operated Cl^−^ channels reverse from excitatory efflux to inhibitory influx during maturation.^43^ Overall Ca^2+^ levels are limited by plasma membrane Ca^2+^ ATPase (*PMCA*). Known drug interactions are indicated in green.

A common problem with many ‘pathway analysis' approaches is that sufficiently many inconsistent findings may be present in the published literature for at least some pathways to fit (almost) any set of genes generated by GW screening. Hence, a major strength of the current study is that the primary hypothesis about Ras/Ca^2+^ signaling being involved had been stated *a priori* based on our published CAE results (Figure 2, bottom), increasing confidence in the current ASD results (‘prioritized subset'^42^) and allowing the specificity of the proposed cutoff for study-specific GWS to be discussed.

Of the top 100 genes selected in AGP I and II, 57 and 47 genes, respectively, could be related to Ras/Ca^2+^ signaling (Supplementary Table 1 and Figure 1), matching a targeted false discovery rate of 50%.^37^ The increasing enrichment toward the top 50 and top 20 genes (Supplementary Table 1), reaching 100% for the top twelve regions in AGP I, attests to the high specificity of the results. Additional support comes from the replication of the results in two independent populations (see below). In an unrelated autoimmune disease, psoriasis, in contrast, the majority of genes identified were located in the HLA region or interleukins (data not shown). The lack of overlap between these unrelated diseases further attests to the specificity of the proposed approach.

To guide with interpretation, the subset of genes among the top 100 reported in our CAE study^20^ that let to the Ras/Ca^2+^ hypothesis (Figure 2, bottom) and the matching genes among the top 100 genes from either of the two stages of the current study are arranged in Figure 2 around a putative ‘consolidated pathway' derived from several ‘canonical pathways'. Although many variations of such a consolidated pathway could be constructed, we contend that there is sufficient consensus among canonical pathways for genes in close proximity to be functionally related.

### Replication across independent populations

With complex diseases, independent studies are not expected to show more than the functional equivalence seen in the close overlap between stages in Figure 2 (top and center). When hundreds of genes contribute,^44^ few, if any, would be expected to be among the most significant in any two independent studies, even in the absence of selection and ascertainment bias. The two AGP populations, however, were collected consecutively in different sets of locations. Female cases could only be included in AGP I, due to imbalances in disease severity and chip platform usage om AGP II. While of limited value for confirming the Ras/Ca^2+^ signaling hypothesis, the results of the exploratory pathway analysis (Supplementary Table 2) suggest that AGP I and II patients vary more with respect to behavior (‘schizophrenia') and developmental risk factors (‘neuritogenesis'), respectively.

Still, seven genes among the top 100 in both stages (Table 1c) can be directly related to the hypothesized pathway (ranks and Fisher's^45^ combined *s*-values *s*_F_ in parentheses): *SORCS2* (10th/36th, 9.85) binds *NGFR* and mediates apoptosis^46^ as well as responses to proneurotrophins.^47^ *CDH13* (45th/25th, 8.86) is an atypical cadherin involved in cell signaling, rather than adhesion. It colocalizes with α_v_β_3_ integrin,^48^ downregulates neural cell growth^49^ and was disrupted by a microdeletion in an ASD case.^50^ The membrane progesterone receptor^36^ *PGR* (66th/18th, 8.78) drives ERK/MAPK signaling^51^ and contributes to neuron excitability through steroids^52^ in the brain.^53^ *GRK5* (36th/59th, 8.68) controls neuronal morphogenesis^54^ by phosphorylating G-protein-coupled receptors (GPCRs) and initiates β-arrestin-mediated downregulation in a Ca^2+^/calmodulin-dependent manner. *PZP* (34th/93rd, 8.56) interacts with the target of minocycline, *MMP9*,^55^ which cleaves^56^ the extracellular component of *CD44*,^57^ whose expression has been implicated in ASD^58^ and whose intracellular component interacts with *RAS*^59^ via both *ERBB2* and *PLC*.^60,61^ *PTPRT* (90th/20th, 8.57) will be discussed below.

Among the top genes with *s*_F_ > 8.5 (Table 1d) are several more ASD-related genes. *KCNMA1* (795th/1st, 9.38) and *DOK5* (2nd/497th, 9.29), also listed among the individual genes, are a Maxi-K channel in which rare mutations have been identified^62^ and a gene that mediates neurite outgrowth, respectively. *HIVEP2* (110th/4th, 9.22) is also known as *Schnurri-2* and Shn-2^(^^−/−)^ mice exhibited hypersensitivity to stress accompanied by anxiety-like behavior.^63^ Mutations in *SEPT9* (123rd/8th, 9.01) cause hereditary neuralgic amyotrophy.^64^ *DMD* (17th/88th, 8.97) is a member of a glycoprotein complex, which accumulates at a variety of neuronal synapses. Dystrophin is associated with Duchenne and Becker muscular dystrophies (DMD), where it is implicated in signaling events and synaptic transmission. DMD is comorbid to ASD.^65^ Two studies found a genetic association of DMD with ASD,^6,66^ and one deletion in *DMD* was found in a CNV analysis of the AGP I data.^14^ The ortholog of *SHROOM3* (23rd/109th, 8.66) in mice is required for proper neurolation.^67^ The combined results of *PPFIBP1* (11th/197th, 9.17), which is also included in the univariate results above, will be discussed in the context of PTPRs below.

### Functional clusters of genes

Overall, the results (see Supplementary Figure 1 for a Manhattan plot) are highly consistent with previously proposed aspects of the etiology of ASD. The clusters of genes implicated in both of the independent stages (Figure 2a/b) consistently overlap with our published CAE results (Figure 2c), confirming the involvement of ion channels (top right) and signaling downstream of *RAS* (bottom left), with two noticeable additional gene clusters in ASD. Both stages implicate several genes involved in deactivation of growth factor (GF) receptors (Figure 2a/b, top left) as ASD-specific risk factors and chloride (Cl^−^) signaling, either through Ca^2+^ activated Cl^−^ channels (CaCC, Figure 2a, top right) or through the lysosomal Cl^−^/H^+^ exchange transporter *CLCN7* (Figure 2b, top right), whose disruption leads to widespread degeneration in the CNS of mice.^68^ In CAE, in contrast, several genes related to ER and Golgi as well as *DST* interacting with F-actin^69^ support the hypothesis that assembly/trafficking and lateral diffusion, respectively, of GABA_A_ receptors, potentially mediated through *RhoA* and *CDC42,*^70^ may be more specific to the etiology of epilepsies.^71^

### Broad evidence for involvement of PTPRs

One of the most striking observations is the involvement of at least five PTPRs in ASD (Figure 2, 10 o'clock position). PTPRs (Table 1e) regulate GF signaling through reversible protein tyrosine dephosphorylation.^72^ *PTPRT* (90th/20th, 8.57) was implicated in ASD by a deletion^73^ (Table S2 AU018704) and a somatic mutation.^74^ It is the PTPR most frequently mutated in colon cancer, where all five missense mutations identified reduced phosphatase activity.^75^ *PTPRD* (519th/84th, 7.26), for which rare CNVs were previously reported,^14^ and its ligand^76^ *IL1RAPL2* (10th in AGP II), which is associated with X-linked non-syndromic mental retardation, are also implicated. *De novo* disruptions in *PPFIA1* and the neighboring *SHANK2* were recently reported in a person with autistic behavior^77^ and, here, *PTPRF* is implicated through the association of its interacting binding protein 1 *PPFIBP1* (11th/197th, 9.14) and *ERC2* (49th in AGP II). *PTPRG* is known to bind both *CNTN6* (13th in AGP II) and *CNTN4* (837th/78th, 7.06),^78^ which play an important role in postnatal brain development.^79^ *PTPRB* (21st/880th, 7.77) binds *CNTN1*, which is involved in axonal expression and neurite extension.^80^

### Replication of PTPR wide loci across the independent stages

**Figure 3:**
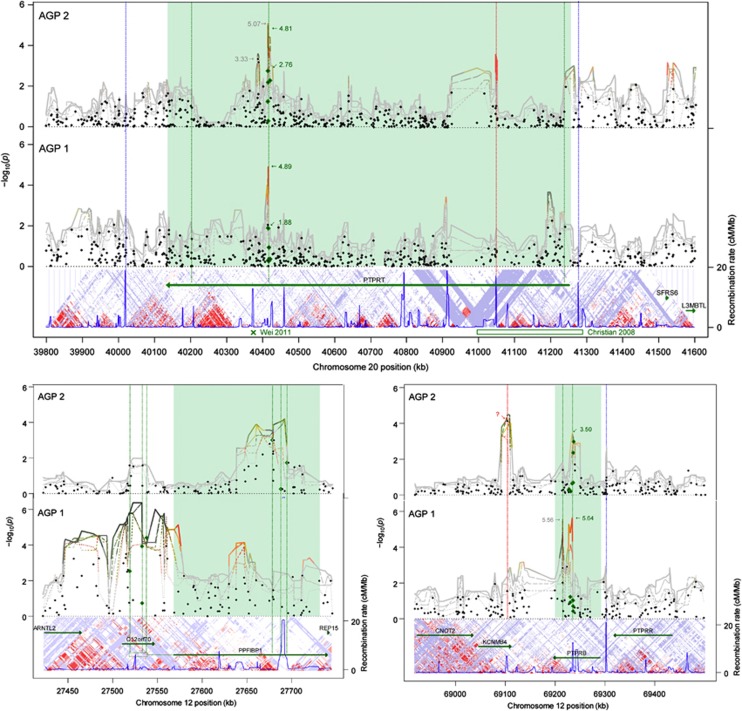
Extended Manhattan plot of μGWAS results for *PTPRT* (top), *PPFIBP1* (bottom/left), and *PTPRB* (bottom/right) by AGP stage. The X-axis shows base pairs within chromosome. Black dots indicate significance in ssGWAS, lines indicate significance in diplotypes of width 2 (dotted) .. 6 (solid), red color indicates low μ-scores for reliability, suggesting a potential artifact, unless supported in both populations. Green dots and s-values indicate univariate results for SNPs within the most significant region. s-Values in *gray* indicate nearby results. Below the panels are gene annotations, LD blocks, and recombination rate from HapMap.^81^ The *PTPRT* region comprises rs6102794, rs6072693, rs6072694, rs6102795, rs6016759 and rs6102798. The ‘x' and box at the bottom indicate a somatic mutation at rs146825584^74^ and a deletion at 41,036,259–41,300,521^73^ (Supplementary Table S2, AU018704), respectively. The *PTPRB*: region comprises rs3782377, rs2567137, rs2567133, rs2278342, rs2116209 and rs2278341. *KCNMB4* results driven by a single SNP in one population only (rs787931, red ‘x') are indicated as a potential artifact, but the related KCNMA1 was the most significant gene in AGP II (Table 1).

Notably, the region of high significance in two of the *PTPRs*, *PTPRT* (Figure 3, top/left) and *PTPRB* (21st/880th, 9.11) (Figure 3, bottom/left), comprises the same SNPs in both independent stages. Moreover, the PTPRT region is located in the same LD block as a known somatic mutation (rs146825584).^74^

### Evidence for PTPR risk being epistatic

To further explore the risk conveyed by PTPRs, we scored male subjects combined with 1047 male controls from a melanoma study genotyped on the same chip platform (see Supplementary Material) stratified by stages,^27^ see Figure 5 (*PTPRT*) and Supplementary Figure 13 (*PTPRB*, *PTPRD*, and *PPFIBP1*). The polarized diplotypes with the best discrimination by stage are highly consistent, indicating that the populations agree not only in the location of the risk factors, but also in the high risk alleles.

For *PTPRT* and *PTPRB* in both stages and for *PTPRD* and *PPFIBP1* in AGP I, SDA and HFA cases scored higher and lower than controls, respectively, so that no difference could have been detected by comparing all cases against controls. This result are consistent with the hypothesis that that PTPR variations, in general, merely affect body size (and, thus, are not selected against), but in the presence of other genetic risk factors contribute significantly to deciding the fate of an ASD case towards either HFA or SDA.

### K^+^ and Cl^−^ ion channels as drug targets

Aside from PTPRs (Figure 2, 10 o'clock) as a risk factor for protracted GF signaling, our results suggest a second functional cluster of genes, involved in Cl^−^ transport and signaling, as specific to ASD (Table 1f). In AGP I, the CaCCs ANO4 and ANO7 scored 1st and 70th, respectively. In AGP II, the lysosome membrane H^+^/Cl^−^ exchange transporter *CLCN7* scored 21st, followed by *CAMK2A*, which regulates ion channels, including anoctamins^82^ (55th), and *LRRC7* (densin-180), which regulates *CAMK2A*^83^ (Figure 2a/b, 2 o'clock). The role of the anoctamins in pathophysiology is not well understood, except that CaCC activity in some neurons is predicted to be excitatory^84^ and to have a role in neuropathic pain or nerve regeneration. More recently, CaCCs have also been suggested as involved in ‘neurite (re)growth'.^85^

**Figure 4:**
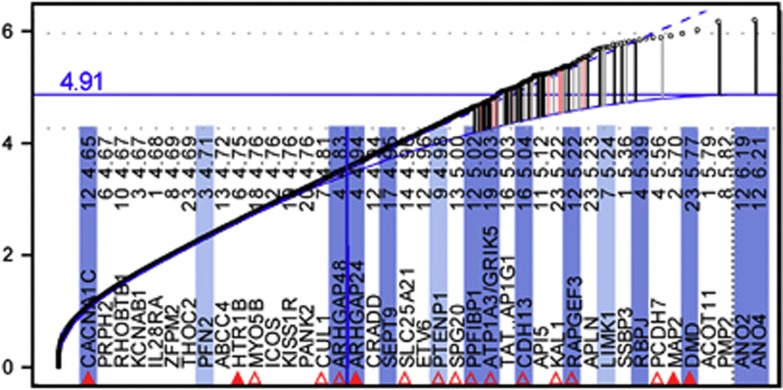
Comparison of HFA cases against all parental controls. Genes shaded in dark and light blue are members of or associated with the Ras/Ca^2+^ pathway (Figure 2), respectively (see Figure 1 for legend). *PFN2* inhibits the formation of IP_3_ and DAG by binding to *PIP2*, *LIMK1* is phosporylated by *ROCK1* and *PAK1*, downstream of *RHOA* and *RAC1*, respectively. *PTENP1* acts as a decoy for *PTEN-targeting* miRNAs.^40^ SLC25A21 may be involved in 2-oxodipate acidemia, which is accompanied by mental retardation and learning disabilities.^41^ Cytogenic bands: ANO2: 12p13.3, ANO4: 12q23.3.

**Figure 5:**
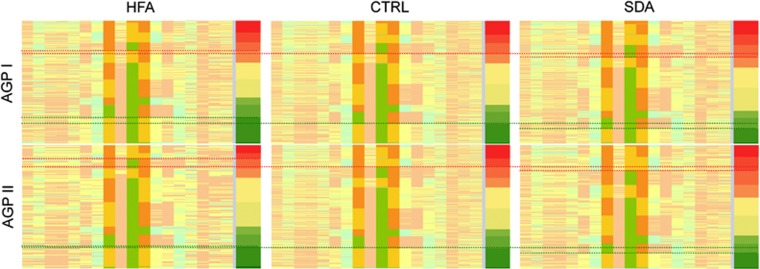
Comparison of *PTPRT* allelotype profiles between SDA cases, melanoma controls, and HFA cases. Rows indicating individual subjects' SNP profile (orange/green: homozygous; yellow: heterozygous) are sorted within each population by diplotype μ-score (dark green to dark read) computed from the three consensus SNPs (rs6102794, rs6072694 and rs6102795 out of the six-SNP *PTPRT* region of Figure 3), which are highlighted as more saturated. Dotted lines are added for visual guidance.

Finally, we compared the HFA and SDA cases as separate groups against all parental controls in the larger AGP I population. Overall, the level of significance is lower and the enrichment is less pronounced, especially for the SDA cases (Supplementary Figure 9), as expected when cases and some controls are related. For the HFA cases (Figure 4, and Supplementary Figure 8), however, a second anoctamin, ANO2, located on the other arm of chromosome 12, competes with ANO4 (Figure 1, left), for the most significant gene among the result. Hence, drugs targeting anoctamins might have broader benefits for the treatment of ASD than in preventing progression to more severe forms of autism.

ANO2 and ANO6 are associated with panic disorder and major depressive disorder, respectively. *ANO3*, *ANO4*, *ANO8* and *ANO10*, but not *ANO1*, are also expressed in neuronal tissue.^86^ As ‘druggable channels', anoctamins ‘may be ideal pharmacological targets to control physiological function or to correct defects in diseases'.^87^ Few drugs, however, target individual anoctamins or even exclusively CaCCs. Cl^−^ channel blockers such as fenamates, for instance, may decrease neuronal excitability primarily by activating Ca^2+^-dependent outward rectifying K^+^ channels.

## Discussion

ASDs are complex diseases involving many genes along common pathways.^23^ Based, in part, on mouse studies^88,89^ and enrichment of CNVs in a previous analysis of the AGP I data,^14^ there is an emerging consensus building that dysregulation of the Ras pathway is involved in ASDs. Ca^2+^ signaling has an excitatory impact on the Ras pathway^90^ and abnormal Ca^2+^ signaling has been implicated in ASD.^91, 92, 93^ Still, ssGWAS have largely failed to elucidate the precise mechanism by which Ras and Ca^2+^ signaling interact and how to determine effective therapies.

While wide-locus GWAS is known to have the potential of higher power over ssGWAS with common diseases,^94,95^ practical problems abound. Many traditional multivariate methods^96^ including simple linear/logistic regression, gene-based^97^ approaches combining ‘individual marker *P*-values' across a gene, and gene-centric^98^ approaches ‘counting the number of minor alleles for each sample at each SNP' assume independence and additivity/multiplicativity of risk factors. As a downside, meaningful non-linear relationships may be overlooked (false negatives), while random errors, not subject to biological constraints, may occasionally fulfill any assumption, so that many ‘significant' results are often false positives due to model misspecification. Increasing degrees of freedom with logistic regression by adding sequential interaction terms for neighboring SNPs may increase likely ‘noise'.^20^

With the advent of mainframe computers, more complex calculations (for example, factor analysis) became feasible. Personal computers triggered the development of resampling methods. Recently, increases in memory to gigabytes and massive parallel computing have spurred the methodological advances making wide-locus GWAS based on a nonparametric approach (*u*-statistics for multivariate data, μGWAS) feasible.^99^ Making only biologically plausible assumptions (additional risk variants within a wide-locus increase risk, albeit to an unknown extent) avoids typical model misspecification biases with traditional methods, whose assumptions (independence and additivity of the risk conferred by the SNPs within a scan statistic window^100^) primarily aim at computational simplicity.^101^

Several alternative strategies have limitations when applied to GWAS of common diseases. Increasing the sample size cannot guard against systematic, though unrelated differences between non-randomized samples taken from outbred populations.^102^ While the power for true disease-related differences increases with sample size, so does the power for equally true selection biases. Analyses based on predefined sets of genes comprising a pathway, including gene-set enrichment^103,104^ have low power when many relevant genes are only indirectly associated with the pathway. One of our most striking observations in this study (and in the previous CAE study) is the lack of association with non-redundant members of the Ras pathway itself, consistent with clinical observations that Rasopathies, such as Costello syndrome, cause more severe phenotypes than most forms of ASD and are routinely selected against. Moreover, our results in both CAE and ASD suggest a role for pseudogenes (*EEF1A1P12* and *PTENP1*, respectively) in support of the previously identified pseudogen *MSNP1*.^7^ Finally, exome sequencing may overlook the variations in introns or promoter regions typical for common diseases^20^ (see Supplementary Figure 3) and the advantage of whole genome (WG) sequencing over GWAS based on SNPs is limited for common, compared to familial risk factors.

Individual *de novo* variations conferring noticeable risk are typically selected against during evolution. Epistatic variations (as originally defined by Fisher^105^) within the same intragenic or promoter region (wide-locus), however, could persist if each variation's contribution *per se* were small. Hence, while principal component analysis can guard against subsets of SNPs related to common ancestry factors, wide-locus GWAS reduces the impact of individual SNPs, in general, and, thus, may guard against a broader range of artifacts. With the risks of artifacts from population stratification reduced as part of the statistical approach, the advantage of family-based association tests (FBAT) to control for population stratification by using hypothetical siblings as controls may pale against the disadvantage of low power in complex diseases,^106,107^ where related subjects are expected to share most, if not all, genetic risk factors. On the other hand, comparisons of cases against unrelated controls could identify risk factors that distinguish ASD cases, in general, from unaffected subjects, which were not present in the AGS population, but the results might more likely be confounded by factors unrelated to the disease (population stratification).

μGWAS increases power by comprehensively analyzing information from several neighboring SNPs, drawing on expected LD from HapMap^81^ and the spatial structure of SNPs within an LD block^20^ without introducing biases through unrealistic assumptions (independence and additivity). The proposed study-specific genome wide significance cutoffs bypass the unattainable goal of guarding against systematic biases in GWAS in favor of guarding against random errors only. Shifting the focus from individual SNPs, which could easily be false positives, to wide loci, which are more likely functional, also shifts the burden to avoid false positives from the decision strategy (increasing the level of significance) to the statistical method (integrating information from neighboring SNPs).

When applied to the AGP data, the consistent results from this hypothesis-driven prioritized subset analysis in two independent populations strongly confirm the Ras/Ca^2+^ hypothesis, and provide, for the first time, evidence-based insights into the etiology of ASD, a novel treatment paradigm, and additional approved drugs that might be repurposed for ASD.

Our results also suggest a reinterpretation of several previously reported findings. The educational attainment study^11^ mentioned in the Introduction, for instance, reported only three apparently unrelated loci reaching conventional GWS: *LRRN2*, *LOC150577*, and *LOC100129158*. Six of the top ten loci, however, point to genes closely related to the Ras/Ca^2+^ pathway (Figure 2): *mir2113* (a micro-RNA located in the same LD block as *LOC100129158*, which has *GRIK2* and *PIK3C2A* among the predicted targets with highest confidence), *PIK3C2B* (in the same LD block as *LRRN2*), *STK24* (containing a *CDC42* binding domain), *ATXN2L* and *ITPR3* (both involved in regulating Ca^2+^ efflux from the ER), and *GPM6A* (involved in *NGF*-dependent Ca^2+^ influx).

Four additional genetic findings relate directly to ASD. First, being able to identify more narrowly defined regions, μGWAS pinpoints *SMAP2*, which encodes a GAP that acts on *ARF1*, a member of a Ras superfamily. Both *SMAP2* and *RIMS3* are located in the same 3.3 Mb region in chromosome 1 (Supplementary Figure 2) which was identified as a microdeletion. *RIMS3* had been selected based on ‘literature review and bioinformatics analyses',^59^ but our results suggest *SMAP2* as the more likely candidate for a gene involved in ASD. Second, μGWAS confirms the involvement of *CNTNAP2*,^108^ with the strongest signal (in AGP II, Supplementary Figure 2) between exons 14 and 15, the same intronic region as rs2710117 previously associated with developmental language disorders^109^ and major depression.^110^ The significant findings in *NXPH1*, *NLGN2*, *DAOA*, and *GRIK5* support its role in ‘localization of potassium channels within differentiating axons' (Figure 2, 1 o'clock), consistent with the rare CNVs seen in *DLGAP2*, *SHANK2*, and *SYNGAP1*,^14^ as well as rare mutations in *NLGN3*.^111^ However, the known functional relationship between *CNTN1*, a binding partner of *PTPRB*, and *CNTN2*,^112,113^ a binding partner of *CNTNAP2*, raises the possibility that *CNTNAP2* may also be involved through its role in ‘mediat[ing] interactions between neurons and glia during nervous system development' (Figure 2, 10 o'clock). Third, ASD has been associated with *HRAS*, although no functional mutation has been identified. In fact, the original publications cautioned that ‘the *TH* and *HRAS-1* genes are molecularly close, might be in linkage disequilibrium, and could reasonably [both] be considered as good candidate genes' for the ‘positive association between autism and two [(3′ and exon 1)] *HRAS* markers'.^114^ Our results suggest that *ANO9*, located within only 100 kb of *HRAS* and in the same LD block might be an even better candidate gene than *TH*, which is located 1.5 Mb and several LD blocks away. Finally, our results are consistent with several of the canonical pathways identified in a previous study,^115^ but also with little overlap among individual SNPs across populations.^116^

Overall, our results strongly suggest that ASD is in large part a neurodevelopmental disease. While it had originally been suggested that symptoms of ASD are present from birth or shortly thereafter,^117^ there is now consensus that symptoms emerge gradually over the first 18 months of life.^118, 119, 120^ Our results lead to a clinical hypothesis testable in a phase II trial for interventions based on a tentative functional interaction between Ras and Ca^2+^ signaling. Our findings are consistent with increased brain volume,^121^ brain connectivity,^122,123^ and skeletal growth correlated with severity of symptoms^124^ and suggest impaired inhibition of neuronal growth due to defects in PTPRs upstream of *RAS* (Figure 2, top left) as a distinctive critical aspect in the etiology of SDA and a hypothesis to ‘elucidate the ‘dark matter' [relating] the [*PI3K*-*AKT*-*mTOR*] pathway'^125^ downstream of *RAS* (Figure 2, bottom left).

The suggested role of PTPR variations in protracted GF signaling suggests the time of accelerated growth during 6–12 months of age for beginning pharmaceutical interventions targeting Ca^2+^ signaling, from the time where a decline in eye fixation and atypical pattern in scanning of faces and social scenes can be observed^120,126,127^ to the time where language regression is seen in some children^128^ at the begin of the ‘stranger anxiety' period,^129^ while interventions targeting downstream *mTOR* signaling^130^ might be most effective when started even earlier, before symptoms are seen.

We posit a counterproductive maladaptive socio-emotional response^131^ to exposure to unfamiliar faces and voices, caused by sensory overload in response to disorganized perception of salient social features, potentially leading to experiences as intolerable as migraines, which are also known to share genetic risk factors,^24^ as a possible explanation for the observed differential shift in attention between unfamiliar and familiar faces^132^ and the hyporesponsiveness to social sensory stimuli in very young children with ASD.^133^ This association might also explain the differences seen in electroencephalography (EEG) measurements at 6–18 months between siblings of ASD children and normal controls.^134^ Structures for ‘social intelligence' may be pruned^135^ in favor of structures for ‘analytical intelligence' during this critical period where connectivity of the brain is validated and refined under the influence of environmental experiences. Over time, the brain adjusts to the over-excitation—as in CAE—so that children outgrow hypersensitivity to social cues before being old enough to report them, but at that time the window of opportunity for developing cortical structures may already be closed.

This reasoning may also explain both the limited success with interventions at later age and the ‘savant skills' in some ASD cases. Along with increased community and clinical awareness of ASD and changing diagnostic standards, demographic shifts from rural to urban environments^136^ and children's increased exposure to television since the 1950s^137^ may have also led to cases of sensory overstimulation in response to social information in children with genetic predisposition and, thus, contributed to the increase in incidence of ASD.^121,138^

**Figure 6:**
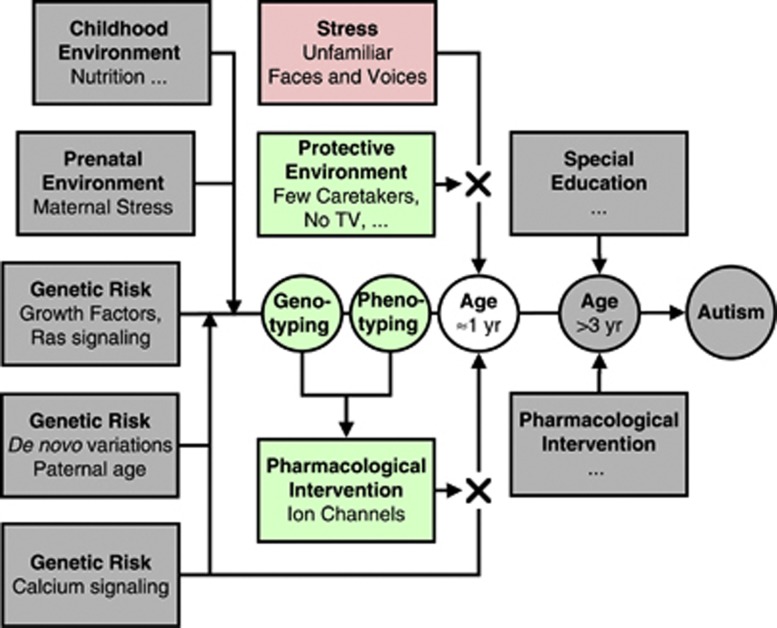
Hypothesized interventions to prevent regression in children with ASD. During the critical period of developing cortical structures for social interactions the risk of stress-induced regression might be reduced through a combination of strategies including a protective environment where exposure to unfamiliar faces is limited and pharmacological interventions to reduce hyperexcitability related to Ca^2+^ signaling by targeting ion channels determined through genetic testing of genes known to be involved in Ca^2+^ signaling among children with a risk phenotype.

This testable clinical hypothesis serves to challenge our current thinking about interventions. A shift in focus may be required from intervention in school aged children to early prevention starting around 12 months of age,^139^ during which time children shape and refine their neural circuitry in response to social stimuli.^140^ Secondly, although the American Academy of Pediatrics' recommendation against television in children under the age of two years^141^ stems from studies in a more general population of children, unfavorable neurodevelopmental and behavioral outcomes in children with ASD might be even more compounded by early media exposure. Furthermore, early behavioral and educational interventions may need to favor personnel familiar to the child (Figure 6).

Our results are consistent with a wide spectrum of genes having mutations contributing to the risk of ASD. Drugs that target ion channels may decrease hyperexcitation to a level where a child does not feel the need to withdraw from social interaction. Testing at marker diplotypes in ion channels or related genes could serve to personalize the choice of the medication most effective in reducing excessive excitation of the Ras pathway during the critical period. Memantine is already used in the treatment of ASD, including in children from 2.5 years of age,^142^ based on a stimulatory effect on neurogenesis in a mouse model for fragile X syndrome (FXS),^143^ the most commonly inherited form of mental retardation, and its limited success in Alzheimer's disease.^144^ Memantine could be most effective in children with mutations in and downstream of NMDA receptors. Gabapentin, approved by the FDA for the treatment of partial seizures in children from 3 years of age^145^ and tested in population-pharmacokinetic studies included subjects starting from age 1 month,^146^ might be repurposed for children with mutations in VOCCs.

Especially for children with mutations involving K^+^ and Cl^−^ signaling, fenamates, which have so far been considered in pain, in general, and in (juvenile) arthritis and (menstrual) migraines^147^ in particular, as well as in epilepsies for decreasing excitatory synaptic activity and reducing neuronal excitability,^148,149^ but not in ASD, might be more effective. Mefenamic acid has been used in preterm children^150^ and, in the EU, is approved for use in infants starting at 6 months of age.^151^ The diuretic bumetanide, which inhibits Cl^−^ influx via the Na^+^–K^+^–2Cl^−^ co-transporter *NKCC1*, has been shown to improve symptoms of ASD in some 3- to 11-year-old children^152^ as well as the efficiency of gabaergic drugs (including barbiturates and benzodiazepines) in neonates,^153^ where intracellular concentration of Cl^−^ is higher than in the mature brain and efflux of Cl^−^ through GABA_A_ receptors is excitatory.^43^ As fenamates target a variety of K^+^ and Cl^−^ channels,^154^ they may have a lower risk for systemic (hypokalaemia^155^) and, in particular, neurodevelopmental^156^ side effects in younger children.

To reduce the duration of exposure during trials, event-related responses to visual stimuli such as familiar and unfamiliar faces, measured by EEG,^134,157^ magnetoencephalography (MEG),^158^ skin conductance,^159^ or eye tracking,^127^ might serve as surrogate endpoints to test the predictive association between genetic risk factors and treatment effectiveness.

Our results attest to a broad spectrum of genetic risk factors contributing to ASD. In particular, other factors than variations in PTPRs might sensitize the Ras pathway to hyperexcitation by interfering with growth factor downregulation. The translational repressor *FMR1*, for instance, directly targets multiple Ras and Ca^2+^ signaling pathway components^160^ and loss of *FMR1* expression may cause FXS through aberrant Ras signaling.^161^ Similarly, reduced repression of mRNA involved in GF signaling might be an alternative mechanism to increase the severity of ASD. Activation of the *PI3K*/*Akt* pathway through mutations in *PTEN*, consistent with our findings from comparing HFA cases against parental controls (*PTENP1*, Figure 4) might be involved in a broader range of the autism spectrum.^162^ The gain-of-expression variation of *MSNP1AS* in ASD cases^7^ is also expected to cause ‘overproliferation' through ‘increased *RhoA* activity',^163^ possibly by competing with miRNAs downregulating MSN,^164^ interacting with the cytoplasmic *CD44*-*ERBB2* complex (Figure 2, 11 o'clock).^61^ Finally, mutations in *SYNGAP1* might restricting the length of time over which *RAS* remains activated.^165^ Hence, while the proposed interventions are unlikely to capture the whole spectrum of risk factors, they might prevent a substantial proportion of children with various risk factors for ASD from developing along the more severe spectrum of this heterogenic disease. The overlap in genetic risk factors between ASD and CAE (Figure 2) suggests another potential benefit of the proposed early intervention. As neonatal seizures *per se* may cause long-term neurological problems,^166^ preventing the postulated intolerable experiences may positively affect a wider range of ASD symptoms. The ability to hone in with μGWAS on specific genetic risk factors in small populations will enable us to develop better diagnostics and to identify subpopulations with other risk factors.

Of course, this multidisciplinary, translational approach is not restricted to ASD, but will further enhance our knowledge of many other complex disorders, thus allowing for the development of a broad range of novel therapeutic modalities with the hope of improved survival and quality of life for many other populations of patients.

Our results suggest that a relevant portion of genetic risk for common diseases is determined through coding variations, as was widely expected after deciphering the human genome ten years ago and could be detected—if GWAS were better adapted to the specifics of common diseases. The paucity of cogent GWAS results in ASD (see the Introduction) compared to some autoimmune and metabolic diseases^167^ may, at least in part, be due to the choice of data source (exome sequencing, rather than whole genome genotyping), study design (comparison against related or hypothetical controls, rather than unrelated cases with different phenotypes), primary outcome (individual SNPs, rather than regions comprising several SNPs), study objective (confirmation of individual SNPs, rather than enrichment of genes along pathways), statistical model (linear model, rather than *u*-statistics), and determination of 'genome-wide significance' cutoffs (WG, rather than selective chromosome estimation). With more appropriate statistical strategies, reanalyzing the data already collected, including data from publicly available repositories (such as the NIH's dbGaP), could finally lead to the insights sought for and the therapies urgently needed.

## Figures and Tables

**Table 1:** Overview of genes in GW analyses meeting the significance criteria and relationships with functional clusters (symbols highlighted in bold) mentioned in the Results section

*s*_*I*_	*s*_*II*_	*s*_*F*_	*Symbol*		*Synonym*	*Entrez*	*Gene name (some shortened to fit)*	*Function (selected from Entrez/UniProtKB/Swiss-Prot/TOCRIS)*
*(a) Genes above the projected apex in ssGWAS (μGWAS P-values shown)*								
6.96	6.21		*ANO4*^a^		*TMEM16D*	121601	*Anoctamin 4*	Ca^2+^-activated Cl^−^ channel (CaCC)
		6.19^b^	*ANO2*^a^		*TMEM16B*	57101	*Anoctamin 2*	Ca^2+^-activated Cl^−^ channel (CaCC)
6.94	3.76	9.29	*DOK5*			55816	*Docking protein 5*	Interacts with phosphorylated receptor tyrosine kinases
5.76			*NTMT1*		*METTL11A*	28989	*N-methyltransferase 1*	Methylates protein targets such as *SET* and *RB*

*(b) Genes above the estimated apex in μGWAS*								
6.81			*MMP10*		Stromelysin 2	4319	*Matrix metallopeptidase 10*	Degrades proteoglycans and fibronectin
6.73			*PDGFB*			5155	*Platelet-derived GF β*	Initiating signaling through the *MAPK*, *PI3K* and *PKCγ* pathways
6.64			*MRGPRX2*			117194	*MAS-related GPR, mbr X2*	G_q_PR mediating cortistatin-stimulated increases in intracellular Ca^2+^
6.63			*SGMS1*		*TMEM23*	259230	*Sphingomyelin synthase 1*	Large, zinc-finger-containing transcription factor
6.62			*GRIK5*			2901	*Glutamate receptor, ionotropic, kainate*	Excitatory neurotransmitter in the CNS
6.59			*SMAP2*			64744	*Small ArfGAP2*	Stromal GTPase-activating protein (GAP) that acts on *ARF1*
6.55			*FAM13A*		*ARHGAP48*	10144	*Fam with sequence similarity 13, mbr A*	Rho GTPase activating protein
6.44	4.84	9.85	*SORCS2*			57537	*Sortilin-related receptor 2*	Containing VPS10 domain
6.38	4.20	9.17	*PPFIBP1*^c^			8496	*Liprin Beta 1*	*PTPRF* interacting protein, binding protein 1
	6.98		*KCNMA1*		*Kca1.1*	3778	*Maxi-K Ca^2+^-activated channel*	Large conductance channel that dampens excitatory events
	6.23		*HTR1B*		*5-HT1B*	3351	*Serotonin receptor 1B*	G-protein-coupled receptor involved in neuropsychiatric disorders

*(c) Genes among the top 100 genes in μGWAS in both stages (in addition to SORCS2)*								
5.27	4.99	8.86	*CDH13*		*H-cadherin*	1012	*Cadherin 13*	Downregulates axon growth during neural differentiation
5.02	5.15	8.78	*PGR*		*NR3C3*	5241	*Progesteron receptor*	Nuclear/membrane^36^ hormone receptor
5.42	4.64	8.68	*GRK5*			2869	*GPR kinase 5*	Phosphorylates the activated forms of GPRs
5.43	4.51	8.56	*PZP*			5858	*Pregnancy-zone protein*	Inhibits all four classes of proteinases
4.89	5.07	8.57	*PTPRT*^c^			11122	*PTP, receptor type, T*	May be involved in signal transduction and cellular adhesion in the CNS

*(d) Additional genes jointly (Fisher) above the joined projected apex in μGWAS (in addition to KCNMA1)*								
4.81	5.81	9.22	*HIVEP2*		*Schnurri-2*	3097	*MHC binding protein 2*	
4.74	5.66	9.01	*SEPT9*			10801	*Septin 9*	Filament-forming cytoskeletal GTPase
5.85	4.52	8.97	*DMD*			1756	*Dystrophin*	Ligand for dystroglycan
5.61	4.44	8.66	*SHROOM3*		*APXL3*	57619	*F-actin-binding protein*	Controls cell shape changes during neural tube closure

*(e) Genes related to PTPRs among the top 100 genes in μGWAS in at least one stage (in addition to PPFIBP1 and PTPRT)*								
5.64	3.47	7.77	*PTPRB*			9665	*PTP, receptor type, B*	Interacts with neuronal receptor, contactin and tenascin C
4.05	4.53	7.26	*PTPRD*			5789	*PTP, receptor type, D*	Interacts with IL1RAPL1 for synapse formation
	5.47		*IL1RAPL2*			26280	*IL-1 receptor accessory protein-like 2*	Closely related to IL1RAPL1
	5.39		*CNTN6*		*NB-3*	27255	*Contactin 6*	Mediates cell surface interactions during NS development
	4.93		*CNTNAP2*		*AUTS15*	26047	*Contactin-associated protein-like 2*	Mediates neuron–glia interactions during NS development
	4.58		*CNTN4*		*BIG-2*	152330	*Contactin 4*	May have a role in the formation of axon connections
	4.68		*ERC2*			26059	*ELKS/RAB6-interacting/CAST fam mbr 2*	May recruit liprin-alpha proteins to the nerve terminals active zone

*(f) Genes related to Cl*^−^*signaling among the top 100 genes in μGWAS in at least one stage (in addition to ANO4)*								
	5.05		*CLCN7*		*PPP1R63*	1186	*Chloride channel, voltage-sensitive 7*	H^+^/Cl^−^ exchange transporter 7
	4.65		*CAMK2A*			815	*CaM dependent kinase II alpha*	Mediates many of the second messenger effects of Ca^2+^
	4.67		*LRRC7*		*Densin-180*	57554	*Leucine-rich repeat containing 7*	Necessary for DISC1 and GRM5 localization to PSD complexes

Abbreviations: Cam, calcium/calmodulin; fam, family; GF, growth factor; GPR, G-protein-coupled receptor; mbr, member; PTP, protein tyrosine phosphatase.

a Genes related to section (f).

b From comparison of HFA cases vs all parental controls, Figure 4.

c Genes related to section (e).

## References

[bib1] Bailey A Le Couteur A Gottesman I Bolton P Simonoff E Yuzda E Autism as a strongly genetic disorder: evidence from a British twin study Psychol Med 1995 25 63 77 7792363 10.1017/s0033291700028099

[bib2] Klei L Sanders SJ Murtha MT Hus V Lowe JK Willsey AJ Common genetic variants, acting additively, are a major source of risk for autism Mol Autism 2012 3 9 23067556 10.1186/2040-2392-3-9 PMC3579743

[bib3] Ozonoff S Young GS Carter A Messinger D Yirmiya N Zwaigenbaum L Recurrence risk for autism spectrum disorders: a Baby Siblings Research Consortium study Pediatrics 2011 128 e488 e495 21844053 10.1542/peds.2010-2825 PMC3164092

[bib4] Devlin B Scherer SW Genetic architecture in autism spectrum disorder Curr Opin Genet Dev 2012 22 229 237 22463983 10.1016/j.gde.2012.03.002

[bib5] Rutter M Changing Concepts and Findings on Autism J Autism Dev Disord 2012 43 1749 1757 23359217 10.1007/s10803-012-1713-7

[bib6] Wang K Zhang H Ma D Bucan M Glessner JT Abrahams BS Common genetic variants on 5p14.1 associate with autism spectrum disorders Nature 2009 459 528 533 19404256 10.1038/nature07999 PMC2943511

[bib7] Kerin T Ramanathan A Rivas K Grepo N Coetzee GA Campbell DB A Noncoding RNA Antisense to Moesin at 5p14.1 in Autism Science Transl Med 2012 4 128ra140 10.1126/scitranslmed.3003479 22491950

[bib8] Ma D Salyakina D Jaworski JM Konidari I Whitehead PL Andersen AN A genome-wide association study of autism reveals a common novel risk locus at 5p14.1 Ann Hum Genet 2009 73 ((Pt 3 263 273 19456320 10.1111/j.1469-1809.2009.00523.x PMC2918410

[bib9] Weiss LA Arking DE A genome-wide linkage and association scan reveals novel loci for autism Nature 2009 461 802 808 19812673 10.1038/nature08490 PMC2772655

[bib10] Smoller JW Craddock N Kendler K Lee PH Neale BM Nurnberger JI Identification of risk loci with shared effects on five major psychiatric disorders: a genome-wide analysis Lancet 2013 381 1371 1379 23453885 10.1016/S0140-6736(12)62129-1 PMC3714010

[bib11] Rietveld CA Medland SE Derringer J Yang J Esko T Martin NW GWAS of 126,559 individuals identifies genetic variants associated with educational attainment Science 2013 340 1467 1471 23722424 10.1126/science.1235488 PMC3751588

[bib12] Anney R Klei L Pinto D Almeida J Bacchelli E Baird G Individual common variants exert weak effects on the risk for autism spectrum disorderspi Hum Mol Genet 2012 21 4781 4792 22843504 10.1093/hmg/dds301 PMC3471395

[bib13] Arking DE Cutler DJ Brune CW Teslovich TM West K Ikeda M A Common Genetic Variant in the Neurexin Superfamily Member CNTNAP2 Increases Familial Risk of Autism Am J Hum Genet 2008 82 160 164 18179894 10.1016/j.ajhg.2007.09.015 PMC2253968

[bib14] Pinto D Pagnamenta AT Klei L Anney R Merico D Regan R Functional impact of global rare copy number variation in autism spectrum disorders Nature 2010 466 368 372 20531469 10.1038/nature09146 PMC3021798

[bib15] Filges I Rothlisberger B Blattner A Boesch N Demougin P Wenzel F Deletion in Xp22.11: PTCHD1 is a candidate gene for X-linked intellectual disability with or without autism Clin Genet 2011 79 79 85 21091464 10.1111/j.1399-0004.2010.01590.x

[bib16] Braunschweig D Krakowiak P Duncanson P Boyce R Hansen RL Ashwood P Autism-specific maternal autoantibodies recognize critical proteins in developing brain Transl Psychiatry 2013 3 e277 23838888 10.1038/tp.2013.50 PMC3731784

[bib17] Howerton CL Morgan CP Fischer DB Bale TL O-GlcNAc transferase (OGT) as a placental biomarker of maternal stress and reprogramming of CNS gene transcription in development Proc Natl Acad Sci USA 2013 110 5169 5174 23487789 10.1073/pnas.1300065110 PMC3612602

[bib18] van Balkom ID Bresnahan M Vuijk PJ Hubert J Susser E Hoek HW Paternal age and risk of autism in an ethnically diverse, non-industrialized setting: Aruba PLoS One 2012 7 e45090 22984615 10.1371/journal.pone.0045090 PMC3439376

[bib19] Puleo CM Schmeidler J Reichenberg A Kolevzon A Soorya LV Buxbaum JD Advancing paternal age and simplex autism Autism 2012 16 367 380 22180389 10.1177/1362361311427154

[bib20] Wittkowski KM Sonakya V Song T Seybold MP Keddache M Durner M From single-SNP to wide-locus: genome-wide association studies identifying functionally related genes and intragenic regions in small sample studies Pharmacogenomics 2013 14 391 401 23438886 10.2217/pgs.13.28 PMC3643309

[bib21] Vorstman JA Ophoff RA Genetic causes of developmental disorders Curr Opin Neurol 2013 26 128 136 23429547 10.1097/WCO.0b013e32835f1a30

[bib22] Levisohn PM The autism-epilepsy connection Epilepsia 2007 48 ((Suppl 9 33 35 18047599 10.1111/j.1528-1167.2007.01399.x

[bib23] van Bokhoven H Genetic and epigenetic networks in intellectual disabilities Ann Rev Genet 2011 45 81 104 21910631 10.1146/annurev-genet-110410-132512

[bib24] Gargus JJ Genetic calcium signaling abnormalities in the central nervous system: seizures, migraine, and autism Ann N Y Acad Sci 2009 1151 133 156 19154521 10.1111/j.1749-6632.2008.03572.x

[bib25] Anney R Klei L Pinto D Regan R Conroy J Magalhaes TR A genome-wide scan for common alleles affecting risk for autism Hum Mol Genet 2010 19 4072 4082 20663923 10.1093/hmg/ddq307 PMC2947401

[bib26] Hoeffding W A class of statistics with asymptotically normal distribution Ann Math Stat 1948 19 293 325

[bib27] Wittkowski KM Friedman-type statistics and consistent multiple comparisons for unbalanced designs J Am Statist Assoc 1988 83 1163 1170

[bib28] Bonferroni CE Teoria statistica delle classi e calcolo delle probabilità Pubblicazioni del Istituto Superiore di Scienze Economiche e Commerciali di Firenze 1936 8 3 62

[bib29] Pearson TA Manolio TA How to interpret a genome-wide association study JAMA 2008 299 1335 1344 18349094 10.1001/jama.299.11.1335

[bib30] Cleveland WS Devlin SJ Locally weighted regression: an approach to regression analysis by local fitting J Am Statist Assoc 1988 83 596 610

[bib31] Tukey JW We need both exploratory and confirmatory American Statistician 1980 34 23 25

[bib32] Fisher RAStatistical Methods and Scientific Inference, Hafner: New York, 1956.

[bib33] Gigerenzer G Mindless statistics J Soc Econ 2003 33 587 606

[bib34] Tukey JW Exploratory Data analysis Reading, MAAddison-Wesley

[bib35] Tukey JW Future of Data-Analysis Ann Math Statistics 1962 33 1 67

[bib36] Thomas P Pang Y Membrane progesterone receptors: evidence for neuroprotective, neurosteroid signaling and neuroendocrine functions in neuronal cells Neuroendocrinology 2012 96 162 171 22687885 10.1159/000339822 PMC3489003

[bib37] Schork AJ Thompson WK Pham P Torkamani A Roddey JC Sullivan PF All SNPs are not created equal: genome-wide association studies reveal a consistent pattern of enrichment among functionally annotated SNPs PLoS Genet 2013 9 e1003449 23637621 10.1371/journal.pgen.1003449 PMC3636284

[bib38] Bechhofer RE A single-sample multiple decision procedure for ranking means of normal populations with known variances Ann Math Stat 1954 25 16 39

[bib39] Lehmann EL Some model I problems of selection Ann Math Stat 1961 32 990 1012

[bib40] Poliseno L Salmena L Zhang J Carver B Haveman WJ Pandolfi PP A coding-independent function of gene and pseudogene mRNAs regulates tumour biology Nature 2010 465 1033 1038 20577206 10.1038/nature09144 PMC3206313

[bib41] Fiermonte G Dolce V Palmieri L Ventura M Runswick MJ Palmieri F Identification of the human mitochondrial oxodicarboxylate carrier. Bacterial expression, reconstitution, functional characterization, tissue distribution, and chromosomal location J Biol Chem 2001 276 8225 8230 11083877 10.1074/jbc.M009607200

[bib42] Li C Li M Lange EM Watanabe RM Prioritized subset analysis: improving power in genome-wide association studies Hum Hered 2008 65 129 141 17934316 10.1159/000109730 PMC2858373

[bib43] Ben-Ari Y Excitatory actions of gaba during development: the nature of the nurture Nat Rev Neurosci 2002 3 728 739 12209121 10.1038/nrn920

[bib44] Yu TW Chahrour MH Coulter ME Jiralerspong S Okamura-Ikeda K Ataman B Using whole-exome sequencing to identify inherited causes of autism Neuron 2013 77 259 273 23352163 10.1016/j.neuron.2012.11.002 PMC3694430

[bib45] Fisher RA Combining independent tests of significance The American Statistician 1948 2 30

[bib46] Teng KK Felice S Kim T Hempstead BL Understanding proneurotrophin actions: Recent advances and challenges Dev Neurobiol 2010 70 350 359 20186707 10.1002/dneu.20768 PMC3063094

[bib47] Lane RF St George-Hyslop P Hempstead BL Small SA Strittmatter SM Gandy S Vps10 family proteins and the retromer complex in aging-related neurodegeneration and diabetes J Neurosci 2012 32 14080 14086 23055476 10.1523/JNEUROSCI.3359-12.2012 PMC3576841

[bib48] Berx G van Roy F Involvement of members of the cadherin superfamily in cancer Cold Spring Harb Perspect Biol 2009 1 a003129 20457567 10.1101/cshperspect.a003129 PMC2882122

[bib49] Takeuchi T Misaki A Liang SB Tachibana A Hayashi N Sonobe H Expression of T-cadherin (CDH13, H-Cadherin) in human brain and its characteristics as a negative growth regulator of epidermal growth factor in neuroblastoma cells J Neurochem 2000 74 1489 1497 10737605 10.1046/j.1471-4159.2000.0741489.x

[bib50] Sanders SJ Ercan-Sencicek AG Hus V Luo R Murtha MT Moreno-De-Luca D Multiple recurrent de novo CNVs, including duplications of the 7q11.23 Williams syndrome region, are strongly associated with autism Neuron 2011 70 863 885 21658581 10.1016/j.neuron.2011.05.002 PMC3939065

[bib51] Boonyaratanakornkit V Scott MP Ribon V Sherman L Anderson SM Maller JL Progesterone receptor contains a proline-rich motif that directly interacts with SH3 domains and activates c-Src family tyrosine kinases Mol Cell 2001 8 269 280 11545730 10.1016/s1097-2765(01)00304-5

[bib52] Losel R Wehling M Nongenomic actions of steroid hormones Nat Rev Mol Cell Biol 2003 4 46 56 12511868 10.1038/nrm1009

[bib53] Brinton RD Thompson RF Foy MR Baudry M Wang J Finch CE Progesterone receptors: form and function in brain Front Neuroendocrinol 2008 29 313 339 18374402 10.1016/j.yfrne.2008.02.001 PMC2398769

[bib54] Chen Y Wang F Long H Wu Z Ma L GRK5 promotes F-actin bundling and targets bundles to membrane structures to control neuronal morphogenesis J Cell Biol 2011 194 905 920 21930777 10.1083/jcb.201104114 PMC3207290

[bib55] Arbelaez LF Bergmann U Tuuttila A Shanbhag VP Stigbrand T Interaction of matrix metalloproteinases-2 and -9 with pregnancy zone protein and alpha2-macroglobulin Arch Biochem Biophys 1997 347 62 68 9344465 10.1006/abbi.1997.0309

[bib56] Chetty C Vanamala SK Gondi CS Dinh DH Gujrati M Rao JS MMP-9 induces CD44 cleavage and CD44 mediated cell migration in glioblastoma xenograft cells Cell Signal 2012 24 549 559 22024282 10.1016/j.cellsig.2011.10.008 PMC3481542

[bib57] Peng ST Su CH Kuo CC Shaw CF Wang HS CD44 crosslinking-mediated matrix metalloproteinase-9 relocation in breast tumor cells leads to enhanced metastasis Int J Oncol 2007 31 1119 1126 17912438

[bib58] Hu VW Frank BC Heine S Lee NH Quackenbush J Gene expression profiling of lymphoblastoid cell lines from monozygotic twins discordant in severity of autism reveals differential regulation of neurologically relevant genes BMC Genomics 2006 7 118 16709250 10.1186/1471-2164-7-118 PMC1525191

[bib59] Kumar RA Sudi J Babatz TD Brune CW Oswald D Yen M A de novo 1p34.2 microdeletion identifies the synaptic vesicle gene RIMS3 as a novel candidate for autism J Med Genet 2010 47 81 90 19546099 10.1136/jmg.2008.065821 PMC2921284

[bib60] Cichy J Pure E The liberation of CD44 J Cell Biol 2003 161 839 843 12796473 10.1083/jcb.200302098 PMC2172964

[bib61] Zöller M CD44: can a cancer-initiating cell profit from an abundantly expressed molecule Nat Rev Cancer 2011 11 254 267 21390059 10.1038/nrc3023

[bib62] Laumonnier F Roger S Guerin P Molinari F M'Rad R Cahard D Association of a functional deficit of the BKCa channel, a synaptic regulator of neuronal excitability, with autism and mental retardation Am J Psychiatry 2006 163 1622 1629 16946189 10.1176/ajp.2006.163.9.1622

[bib63] Takagi T Jin W Taya K Watanabe G Mori K Ishii S Schnurri-2 mutant mice are hypersensitive to stress and hyperactive Brain Res 2006 1108 88 97 16836985 10.1016/j.brainres.2006.06.018

[bib64] Kuhlenbaumer G Hannibal MC Nelis E Schirmacher A Verpoorten N Meuleman J Mutations in SEPT9 cause hereditary neuralgic amyotrophy Nat Genet 2005 37 1044 1046 16186812 10.1038/ng1649

[bib65] Wu JY Kuban KC Allred E Shapiro F Darras BT Association of Duchenne muscular dystrophy with autism spectrum disorder J Child Neurol 2005 20 790 795 16417872 10.1177/08830738050200100201

[bib66] Chung RH Ma D Wang K Hedges DJ Jaworski JM Gilbert JR An X chromosome-wide association study in autism families identifies TBL1X as a novel autism spectrum disorder candidate gene in males Mol Autism 2011 3 2 10.1186/2040-2392-2-18 PMC3305893 22050706

[bib67] Hildebrand JD Soriano P Shroom, a PDZ domain-containing actin-binding protein, is required for neural tube morphogenesis in mice Cell 1999 99 485 497 10589677 10.1016/s0092-8674(00)81537-8

[bib68] Kasper D Planells-Cases R Fuhrmann JC Scheel O Zeitz O Ruether K Loss of the chloride channel ClC-7 leads to lysosomal storage disease and neurodegeneration EMBO J 2005 24 1079 1091 15706348 10.1038/sj.emboj.7600576 PMC554126

[bib69] Ryan SD Bhanot K Ferrer A De Repentigny Y Chu A Microtubule stability, Golgi organization, and transport flux require dystonin-a2–MAP1B interaction J Cell Biol 2012 196 727 742 22412020 10.1083/jcb.201107096 PMC3308695

[bib70] Chi X Wang S Huang Y Stamnes M Chen JL Roles of Rho GTPases in intracellular transport and cellular transformation Int J Mol Sci 2013 14 7089 7108 23538840 10.3390/ijms14047089 PMC3645678

[bib71] Jacob TC Moss SJ Jurd R GABAA receptor trafficking and its role in the dynamic modulation of neuronal inhibition Nature Rev Neurosci 2008 9 331 343 18382465 10.1038/nrn2370 PMC2709246

[bib72] Tonks NK Protein tyrosine phosphatases: from genes, to function, to disease Nat Rev Mol Cell Biol 2006 7 833 846 17057753 10.1038/nrm2039

[bib73] Christian SL Brune CW Sudi J Kumar RA Liu S Karamohamed S Novel Submicroscopic Chromosomal Abnormalities Detected in Autism Spectrum Disorder Biological Psychiatry 2008 63 1111 1117 18374305 10.1016/j.biopsych.2008.01.009 PMC2440346

[bib74] Wei X Walia V Lin JC Teer JK Prickett TD Gartner J Exome sequencing identifies GRIN2A as frequently mutated in melanoma Nat Genet 2011 43 442 446 21499247 10.1038/ng.810 PMC3161250

[bib75] Wang Z Shen D Parsons DW Bardelli A Sager J Szabo S Mutational analysis of the tyrosine phosphatome in colorectal cancers Science 2004 304 1164 1166 15155950 10.1126/science.1096096

[bib76] Valnegri P Montrasio C Brambilla D Ko J Passafaro M Sala C The X-linked intellectual disability protein IL1RAPL1 regulates excitatory synapse formation by binding PTPδ and RhoGAP2 Hum Mol Genet 2011 20 4797 4809 21926414 10.1093/hmg/ddr418 PMC3221541

[bib77] Schluth-Bolard C Labalme A Cordier MP Till M Nadeau G Tevissen H Breakpoint mapping by next generation sequencing reveals causative gene disruption in patients carrying apparently balanced chromosome rearrangements with intellectual deficiency and/or congenital malformations J Med Genet 2013 50 144 150 23315544 10.1136/jmedgenet-2012-101351

[bib78] Bouyain S Watkins DJ The protein tyrosine phosphatases PTPRZ and PTPRG bind to distinct members of the contactin family of neural recognition molecules Proc Natl Acad Sci 2010 107 2443 2448 20133774 10.1073/pnas.0911235107 PMC2823867

[bib79] Zuko A Bouyain S van der Zwaag B Burbach JPH Contactins: Structural aspects in relation to developmental functions in brain disease Adv Protein Chem Struct Biol 2011 84 143 180 21846565 10.1016/B978-0-12-386483-3.00001-X PMC9921585

[bib80] Shimoda Y Watanabe K Contactins: emerging key roles in the development and function of the nervous system Cell Adh Migr 2009 3 64 70 19262165 10.4161/cam.3.1.7764 PMC2675151

[bib81] Frazer KA Ballinger DG Cox DR Hinds DA Stuve LL Gibbs RA A second generation human haplotype map of over 3.1 million SNPs Nature 2007 449 851 861 17943122 10.1038/nature06258 PMC2689609

[bib82] Verkman AS Galietta LJV Chloride channels as drug targets Nat Rev Drug Discov 2009 8 153 171 19153558 10.1038/nrd2780 PMC3601949

[bib83] Robison AJ Bass MA Jiao Y MacMillan LB Carmody LC Bartlett RK Multivalent interactions of calcium/calmodulin-dependent protein kinase II with the postsynaptic density proteins NR2B, densin-180, and alpha-actinin-2 J Biol Chem 2005 280 35329 35336 16120608 10.1074/jbc.M502191200

[bib84] Herault J Petit E Martineau J Perrot A Lenoir P Cherpi C Autism and genetics: clinical approach and association study with two markers of HRAS gene Am J Med Genet 1995 60 276 281 7485261 10.1002/ajmg.1320600404

[bib85] Boudes M Scamps F Calcium-activated chloride current expression in axotomized sensory neurons: what for Front Mol Neurosci 2012 5 35 22461766 10.3389/fnmol.2012.00035 PMC3309971

[bib86] Kunzelmann K Tian Y Martins J Faria D Kongsuphol P Ousingsawat J Anoctamins Pflügers Archiv Eur J Physiol 2011 462 195 208 10.1007/s00424-011-0975-9 21607626

[bib87] Berg J Yang H Jan LY Ca2+-activated Cl- channels at a glance J Cell Sci 2012 125 1367 1371 22526416 10.1242/jcs.093260 PMC3336373

[bib88] Zou H Yu Y Sheikh AM Malik M Yang K Wen G Association of upregulated Ras/Raf/ERK1/2 signaling with autism Genes Brain Behav 2011 10 615 624 21595826 10.1111/j.1601-183X.2011.00702.x

[bib89] Srivastava DP Woolfrey KM Jones KA Anderson CT Smith KR Russell TA An autism-associated variant of Epac2 reveals a role for Ras/Epac2 signaling in controlling basal dendrite maintenance in mice PLoS Biol 2012 10 e1001350 22745599 10.1371/journal.pbio.1001350 PMC3383751

[bib90] Cullen PJ Lockyer PJ Integration of calcium and Ras signalling Nat Rev Mol Cell Biol 2002 3 339 348 11988768 10.1038/nrm808

[bib91] Lainhart JE Bigler ED Bocian M Coon H Dinh E Dawson G Head circumference and height in autism: a study by the Collaborative Program of Excellence in Autism Am J Med Genet A 2006 140 2257 2274 17022081 10.1002/ajmg.a.31465 PMC4899843

[bib92] McCaffery P Deutsch CK Macrocephaly and the control of brain growth in autistic disorders Prog Neurobiol 2005 77 38 56 16280193 10.1016/j.pneurobio.2005.10.005

[bib93] Deutsch CK Joseph RM Brief report: cognitive correlates of enlarged head circumference in children with autism J Autism Dev Disord 2003 33 209 215 12757362 10.1023/a:1022903913547

[bib94] Goldstein DB Common Genetic Variation and Human Traits N Engl J Med 2009 360 1696 1698 19369660 10.1056/NEJMp0806284

[bib95] McCarthy MI Abecasis GR Cardon LR Goldstein DB Little J Ioannidis JP Genome-wide association studies for complex traits: consensus, uncertainty and challenges Nat Rev Genet 2008 9 356 369 18398418 10.1038/nrg2344

[bib96] Ballard DH Cho J Zhao HY Comparisons of Multi-Marker Association Methods to Detect Association Between a Candidate Region and Disease Genet Epidemiol 2009 34 201 212 19810024 10.1002/gepi.20448 PMC3158797

[bib97] Liu JZ McRae AF Nyholt DR Medland SE Wray NR Brown KM A versatile gene-based test for genome-wide association studies Am J Hum Genet 2010 87 139 145 20598278 10.1016/j.ajhg.2010.06.009 PMC2896770

[bib98] Nguyen LB Diskin SJ Capasso M Wang K Diamond MA Glessner J Phenotype Restricted Genome-Wide Association Study Using a Gene-Centric Approach Identifies Three Low-Risk Neuroblastoma Susceptibility Loci PLoS Genet 2011 7 e1002026 21436895 10.1371/journal.pgen.1002026 PMC3060064

[bib99] Morales JF Song T Auerbach AD Wittkowski KM Phenotyping genetic diseases using an extension of μ-scores for multivariate data Stat Appl Genet Mol 2008 7 19 10.2202/1544-6115.1372 18597665

[bib100] Ionita-Laza I Makarov V Buxbaum Joseph D Scan-Statistic Approach Identifies Clusters of Rare Disease Variants in LRP2, a Gene Linked and Associated with Autism Spectrum Disorders, in Three Datasets Am J Hum Genet 2012 90 1002 1013 22578327 10.1016/j.ajhg.2012.04.010 PMC3370275

[bib101] Finkelstein DM Schoenfeld DA Combining mortality and longitudinal measures in clinical trials Stat Med 1999 18 1341 1354 10399200 10.1002/(sici)1097-0258(19990615)18:11<1341::aid-sim129>3.0.co;2-7

[bib102] Meehl PE Theoretical risks and tabular asterisks: Sir Karl, Sir Ronald, and the slow progress of soft psychology J Consulting Clin Psychol 1978 46 806 834

[bib103] Mootha VK Lindgren CM Eriksson KF Subramanian A Sihag S Lehar J PGC-1alpha-responsive genes involved in oxidative phosphorylation are coordinately downregulated in human diabetes Nature Genet 2003 34 267 273 12808457 10.1038/ng1180

[bib104] Subramanian A Tamayo P Mootha V Mukherjee S Ebert B Gillette M Gene set enrichment analysis: A knowledge-based approach for interpreting genome-wide expression profiles Proc Natl Acad Sci USA 2005 102 15545 15550 16199517 10.1073/pnas.0506580102 PMC1239896

[bib105] Fisher RA The correlation between relatives on the supposition of Mendelian inheritance Transact Royal Soc Edinburgh 1918 52 399 433

[bib106] Ionita-Laza I Lee S Makarov V Buxbaum JD Lin X Family-based association tests for sequence data, and comparisons with population-based association tests Eur J Hum Genet 2013 21 1158 1162 23386037 10.1038/ejhg.2012.308 PMC3778346

[bib107] Risch N Teng J The relative power of family-based and case-control designs for linkage disequilibrium studies of complex human diseases I. DNA pooling Genome Res 1998 8 1273 1288 9872982 10.1101/gr.8.12.1273

[bib108] Penagarikano O Geschwind DH What does CNTNAP2 reveal about autism spectrum disorder Trends Mol Med 2012 18 156 163 22365836 10.1016/j.molmed.2012.01.003 PMC3633421

[bib109] Vernes SC Newbury DF Abrahams BS Winchester L Nicod J Groszer M A Functional Genetic Link between Distinct Developmental Language Disorders N Engl J Med 2008 359 2337 2345 18987363 10.1056/NEJMoa0802828 PMC2756409

[bib110] Ji W Li T Pan Y Tao H Ju K Wen Z CNTNAP2 is significantly associated with schizophrenia and major depression in the Han Chinese population Psychiatry Res 2013 207 225 228 23123147 10.1016/j.psychres.2012.09.024

[bib111] Földy C Malenka Robert C Südhof Thomas C Autism-associated neuroligin-3 mutations commonly disrupt tonic endocannabinoid signaling Neuron 2013 78 498 509 23583622 10.1016/j.neuron.2013.02.036 PMC3663050

[bib112] Buttiglione M Revest JM Pavlou O Karagogeos D Furley A Rougon G A functional interaction between the neuronal adhesion molecules TAG-1 and F3 modulates neurite outgrowth and fasciculation of cerebellar granule cells J Neurosci 1998 18 6853 6870 9712656 10.1523/JNEUROSCI.18-17-06853.1998 PMC6792965

[bib113] Pavlou O Theodorakis K Falk J Kutsche M Schachner M Faivre-Sarrailh C Analysis of interactions of the adhesion molecule TAG-1 and its domains with other immunoglobulin superfamily members Mol Cell Neurosci 2002 20 367 381 12139915 10.1006/mcne.2002.1105

[bib114] Herault J Perrot A Barthelemy C Buchler M Cherpi C Leboyer M Possible association of c-Harvey-Ras-1 (HRAS-1) marker with autism Psychiatry Res 1993 46 261 267 8098541 10.1016/0165-1781(93)90094-w

[bib115] Skafidas E Testa R Zantomio D Chana G Everall IP Pantelis C Predicting the diagnosis of autism spectrum disorder using gene pathway analysis Mol Psychiatry 2012 10.1038/mp.2012.126 PMC3966080 22965006

[bib116] Robinson EB Howrigan D Yang J Ripke S Anttila V Duncan LE Response to 'Predicting the diagnosis of autism spectrum disorder using gene pathway analysis' Mol Psychiatry 2013 504 427 431 10.1038/mp.2013.125 PMC4113933 24145379

[bib117] Kanner L Autistic disturbances of affective contact Nervous Child 1943 2 217 250 4880460

[bib118] Ozonoff S Iosif AM Baguio F Cook IC Hill MM Hutman T A prospective study of the emergence of early behavioral signs of autism J Am Acad Child Adolesc Psychiatry 2010 49 256 266 20410715 PMC2923050

[bib119] Courchesne E Brain development in autism: Early overgrowth followed by premature arrest of growth Ment Retard Dev Disabil Res Rev 2004 10 106 111 15362165 10.1002/mrdd.20020

[bib120] Jones W Klin A Attention to eyes is present but in decline in 2-6-month-old infants later diagnosed with autism Nature 2013 504 427 431 24196715 10.1038/nature12715 PMC4035120

[bib121] Hazlett HC Poe MD Gerig G Styner M Chappell C Smith RG Early brain overgrowth in autism associated with an increase in cortical surface area before age 2 years Arch Gen Psychiatry 2011 68 467 476 21536976 10.1001/archgenpsychiatry.2011.39 PMC3315057

[bib122] Supekar K Uddin Lucina Q Khouzam A Phillips J Gaillard William D Kenworthy Lauren E Brain Hyperconnectivity in Children with Autism and its Links to Social Deficits Cell Reports 2013 738 747 24210821 10.1016/j.celrep.2013.10.001 PMC3894787

[bib123] Keown Christopher L Shih P Nair A Peterson N Mulvey Mark E Müller R-A Local Functional Overconnectivity in Posterior Brain Regions Is Associated with Symptom Severity in Autism Spectrum Disorders Cell Reports 2013 5 567 572 24210815 10.1016/j.celrep.2013.10.003 PMC5708538

[bib124] Chawarska K Campbell D Chen L Shic F Klin A Chang J Early generalized overgrowth in boys with autism Arch Gen Psychiatry 2011 68 1021 1031 21969460 10.1001/archgenpsychiatry.2011.106 PMC4878118

[bib125] Berg JM Geschwind DH Autism genetics: searching for specificity and convergence Genome Biol 2012 13 247 22849751 10.1186/gb-2012-13-7-247 PMC3491377

[bib126] Shic F Macari S Chawarska K Speech disturbs face scanning in 6-month-old infants who develop autism spectrum disorder Biol Psychiatry 2014 75 231 237 23954107 10.1016/j.biopsych.2013.07.009 PMC3864607

[bib127] Chawarska K Macari S Shic F Decreased spontaneous attention to social scenes in 6-month-old infants later diagnosed with autism spectrum disorders Biol Psychiatry 2013 74 195 203 23313640 10.1016/j.biopsych.2012.11.022 PMC3646074

[bib128] Ozonoff S Iosif AM Young GS Hepburn S Thompson M Colomby C Onset patterns in autism: correspondence between home video and parent report J Am Acad Child Adolesc Psychiatry 2011 50 796 806 21784299 10.1016/j.jaac.2011.03.012 PMC3668453

[bib129] Jaffe J Beebe B Feldstein S Crown CL Jasnow MD Rhythms of dialogue in infancy: coordinated timing in development Monogr Soc Res Child Dev 2001 66 1 132 11428150

[bib130] Ehninger D Silva AJ Rapamycin for treating Tuberous sclerosis and Autism spectrum disorders Trends Mol Med 2011 17 78 87 21115397 10.1016/j.molmed.2010.10.002 PMC3075964

[bib131] Pfaff DW Rapin I Goldman S Male predominance in autism: neuroendocrine influences on arousal and social anxiety Autism Res 2011 4 163 176 21465671 10.1002/aur.191

[bib132] Luyster RJ Wagner JB Vogel-Farley V Tager-Flusberg H Nelson CA 3rd Neural correlates of familiar and unfamiliar face processing in infants at risk for autism spectrum disorders Brain Topogr 2011 24 220 228 21442325 10.1007/s10548-011-0176-z PMC3171602

[bib133] Baranek GT Watson LR Boyd BA Poe MD David FJ McGuire L Hyporesponsiveness to social and nonsocial sensory stimuli in children with autism, children with developmental delays, and typically developing children Dev Psychopathol 2013 25 307 320 23627946 10.1017/S0954579412001071 PMC3641693

[bib134] Bosl W Tierney A Tager-Flusberg H Nelson C EEG complexity as a biomarker for autism spectrum disorder risk BMC Med 2011 9 18 21342500 10.1186/1741-7015-9-18 PMC3050760

[bib135] Low LK Cheng H-J Axon pruning: an essential step underlying the developmental plasticity of neuronal connections Philos T R Soc London 2006 361 1531 1544 10.1098/rstb.2006.1883 PMC1664669 16939973

[bib136] Rosenberg RE Daniels AM Law JK Law PA Kaufmann WE Trends in autism spectrum disorder diagnoses: 1994-2007 J Autism Dev Disord 2009 39 1099 1111 19294498 10.1007/s10803-009-0723-6

[bib137] Waldman M Nicholsonm S Adilov N Does television cause autism? Working PaperNational Bureau of Economic Research: Cambridge, MA, 2006..

[bib138] Rutter M Incidence of autism spectrum disorders: Changes over time and their meaning* Acta Pædiatrica 2005 94 2 15 10.1111/j.1651-2227.2005.tb01779.x 15858952

[bib139] Crais ER Watson LR Challenges and opportunities in early identification and intervention for children at-risk for autism spectrum disorders 2014 17 Epub Dec 13 2013 10.3109/17549507.2013.862860 24328367

[bib140] Siller SS Broadie K Matrix metalloproteinases and minocycline: therapeutic avenues for fragile X syndrome Neural Plast 2012 2012 124548 22685676 10.1155/2012/124548 PMC3364018

[bib141] American Academy of Pediatrics Council on Communications and Media. Policy Statement Media Use by Children Younger Than 2 Years Pediatrics 2011 128 1040 1045 22007002 10.1542/peds.2011-1753

[bib142] Hosenbocus S Chahal R Memantine: a review of possible uses in child and adolescent psychiatry J Can Acad Child Adolesc Psychiatry 2013 22 166 171 23667364 PMC3647634

[bib143] Larsen AM Bunch L Medicinal chemistry of competitive kainate receptor antagonists ACS Chem Neurosci 2011 2 60 74 22778857 10.1021/cn1001039 PMC3369727

[bib144] Yang Z Zhou X Zhang Q Effectiveness and Safety of Memantine Treatment for Alzheimer's Disease J Alzheimers Dis 2013 36 445 458 23635410 10.3233/JAD-130395

[bib145] Parke-Davis Medication Guide Neurontin Pfizer: New York, NY 2013 32 37

[bib146] Ouellet D Bockbrader HN Wesche DL Shapiro DY Garofalo E Population pharmacokinetics of gabapentin in infants and children Epilepsy Res 2001 47 229 241 11738930 10.1016/s0920-1211(01)00311-4

[bib147] Pringsheim T Davenport WJ Dodick D Acute treatment and prevention of menstrually related migraine headache: evidence-based review Neurology 2008 70 1555 1563 18427072 10.1212/01.wnl.0000310638.54698.36

[bib148] Fernandez M Lao-Peregrin C Martin ED Flufenamic acid suppresses epileptiform activity in hippocampus by reducing excitatory synaptic transmission and neuronal excitability Epilepsia 2010 51 384 390 19732136 10.1111/j.1528-1167.2009.02279.x

[bib149] Yau HJ Baranauskas G Martina M Flufenamic acid decreases neuronal excitability through modulation of voltage-gated sodium channel gating J Physiol 2010 588 ((Pt 20 3869 3882 20724367 10.1113/jphysiol.2010.193037 PMC3000579

[bib150] Ito K Niida Y Sato J Owada E Umetsu M Pharmacokinetics of mefenamic acid in preterm infants with patent ductus arteriosus Acta Paediatr Jpn 1994 36 387 391 7942001 10.1111/j.1442-200x.1994.tb03207.x

[bib151] UK. Public Assessment Report for paediatric studies submitted in accordance with Article 45 of Regulation (EC) No1901/2006, as amended: Mefenamic Acid. The Heads of Medicines Agencies2012

[bib152] Lemonnier E Degrez C Phelep M Tyzio R Josse F Grandgeorge M A randomised controlled trialof bumetanide in the treatment of autism in children Transl Psychiatry 2012 2 e202 23233021 10.1038/tp.2012.124 PMC3565189

[bib153] Dzhala VI Brumback AC Staley KJ Bumetanide enhancesphenobarbital efficacy in a neonatal seizure model Annals of Neurology 2008 63 222 35 17918265 10.1002/ana.21229

[bib154] Greenwood IA Leblanc N Overlapping pharmacology of Ca2+-activated Cl- and K+ channels Trends Pharmacol Sci 2007 28 1 5 17150263 10.1016/j.tips.2006.11.004

[bib155] Ng TM Konopka E Hyderi AF Hshieh S Tsuji Y Kim BJ Comparison of bumetanide- and metolazone-based diuretic regimens to furosemide in acute heart failure J Cardiovasc Pharmacol Ther 2013 18 345 353 23538300 10.1177/1074248413482755

[bib156] Khanna A Walcott BP Kahle KT Limitations of Current GABA Agonists in Neonatal Seizures: Toward GABA Modulation Via the Targeting of Neuronal Cl(-) Transport Front Neurol 2013 4 78 23805124 10.3389/fneur.2013.00078 PMC3691543

[bib157] Elsabbagh M Mercure E Hudry K Chandler S Pasco G Charman T Infant neural sensitivity to dynamic eye gaze is associated with later emerging autism Curr Biol 2012 22 338 342 22285033 10.1016/j.cub.2011.12.056 PMC3314921

[bib158] Yoshimura Y Kikuchi M Ueno S Okumura E Hiraishi H Hasegawa C The Brain's Response to the Human Voice Depends on the Incidence of Autistic Traits in the General Population PLoS One 2013 9 e80126 24278247 10.1371/journal.pone.0080126 PMC3835888

[bib159] Kylliainen A Hietanen JK Skin conductance responses to another person's gaze in children with autism J Autism Dev Disord 2006 36 517 525 16555137 10.1007/s10803-006-0091-4

[bib160] Ascano M Mukherjee N Bandaru P Miller JB Nusbaum JD Corcoran DL FMRP targets distinct mRNA sequence elements to regulate protein expression Nature 2012 492 382 386 23235829 10.1038/nature11737 PMC3528815

[bib161] Hu H Qin Y Bochorishvili G Zhu Y van Aelst L Zhu JJ Ras signaling mechanisms underlying impaired GluR1-dependent plasticity associated with fragile X syndrome J Neurosci 2008 28 7847 7862 18667617 10.1523/JNEUROSCI.1496-08.2008 PMC2553221

[bib162] Napoli E Ross-Inta C Wong S Hung C Fujisawa Y Sakaguchi D Mitochondrial dysfunction in Pten haplo-insufficient mice with social deficits and repetitive behavior: interplay between Pten and p53 PLoS ONE 2012 7 e42504 22900024 10.1371/journal.pone.0042504 PMC3416855

[bib163] McClatchey A Merlin and ERM proteins: unappreciated roles in cancer development Nat Rev Cancer 2003 3 877 883 14668818 10.1038/nrc1213

[bib164] Zhu X Morales FC Agarwal NK Dogruluk T Gagea M Georgescu M-M Moesin is a glioma progression marker that induces proliferation and Wnt/β-catenin pathway activation via interaction with CD44 Cancer Res 2013 73 1142 1155 23221384 10.1158/0008-5472.CAN-12-1040

[bib165] Berryer MH Hamdan FF Klitten LL Moller RS Carmant L Schwartzentruber J Mutations in SYNGAP1 cause intellectual disability, autism, and a specific form of epilepsy by inducing haploinsufficiency Hum Mutat 2013 34 385 394 23161826 10.1002/humu.22248

[bib166] Nardou R Ferrari DC Ben-Ari Y Mechanisms and effects of seizures in the immature brain Semin Fetal Neonatal Med 2013 18 175 184 23702158 10.1016/j.siny.2013.02.003

[bib167] Visscher PM Brown MA McCarthy MI Yang J Five Years of GWAS Discovery Am J Hum Genet 2012 90 7 24 22243964 10.1016/j.ajhg.2011.11.029 PMC3257326

