# Supplementary material for: A novel computational biostatistics approach implies impaired dephosphorylation of growth factor receptors as associated with severity of autism
Source: Transl Psychiatry. 2014 Jan 28;4(1):e354–. doi: 10.1038/tp.2013.124 (PMC3905234; doi:10.1038/tp.2013.124)

Supplementary Information

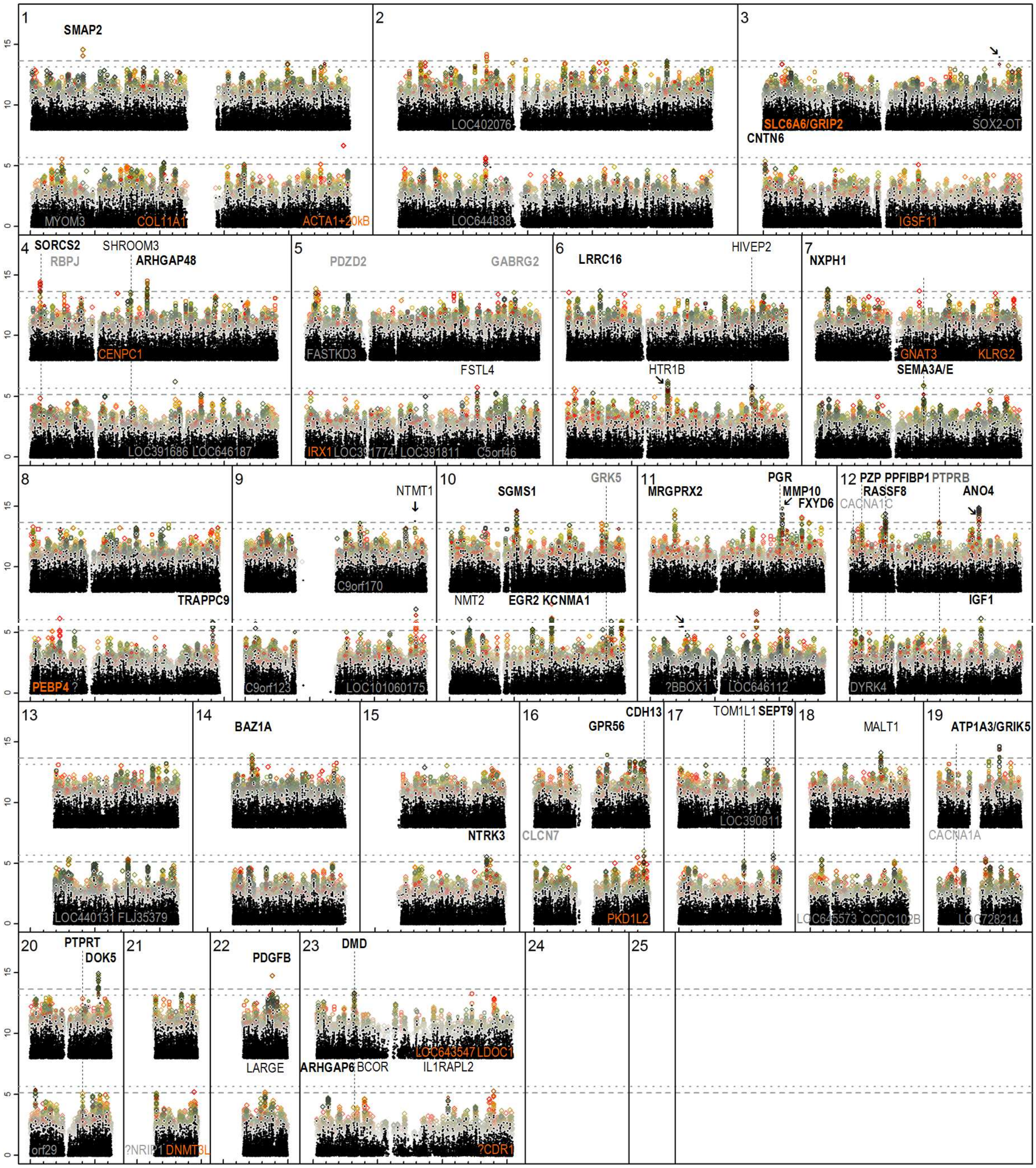

Supplementary Figure 1: μGWAS Manhattan plot. Top: AGP I, bottom: AGP II. The top 20 genes in either Stage are labeled in black, as are the top 11 genes by joint significance (s_+, see Supplementary Table 1, connected by vertical dashed lines). Genes related to the *Ras*/Ca^{2+} pathway are highlighted in bold. Dots in regions primarily significant in male or female cases are shown as squares and circles, respectively. Results shown in red have low reliability (μIC)[20], names in gray indicate additional genes of potential interest, space permitting.

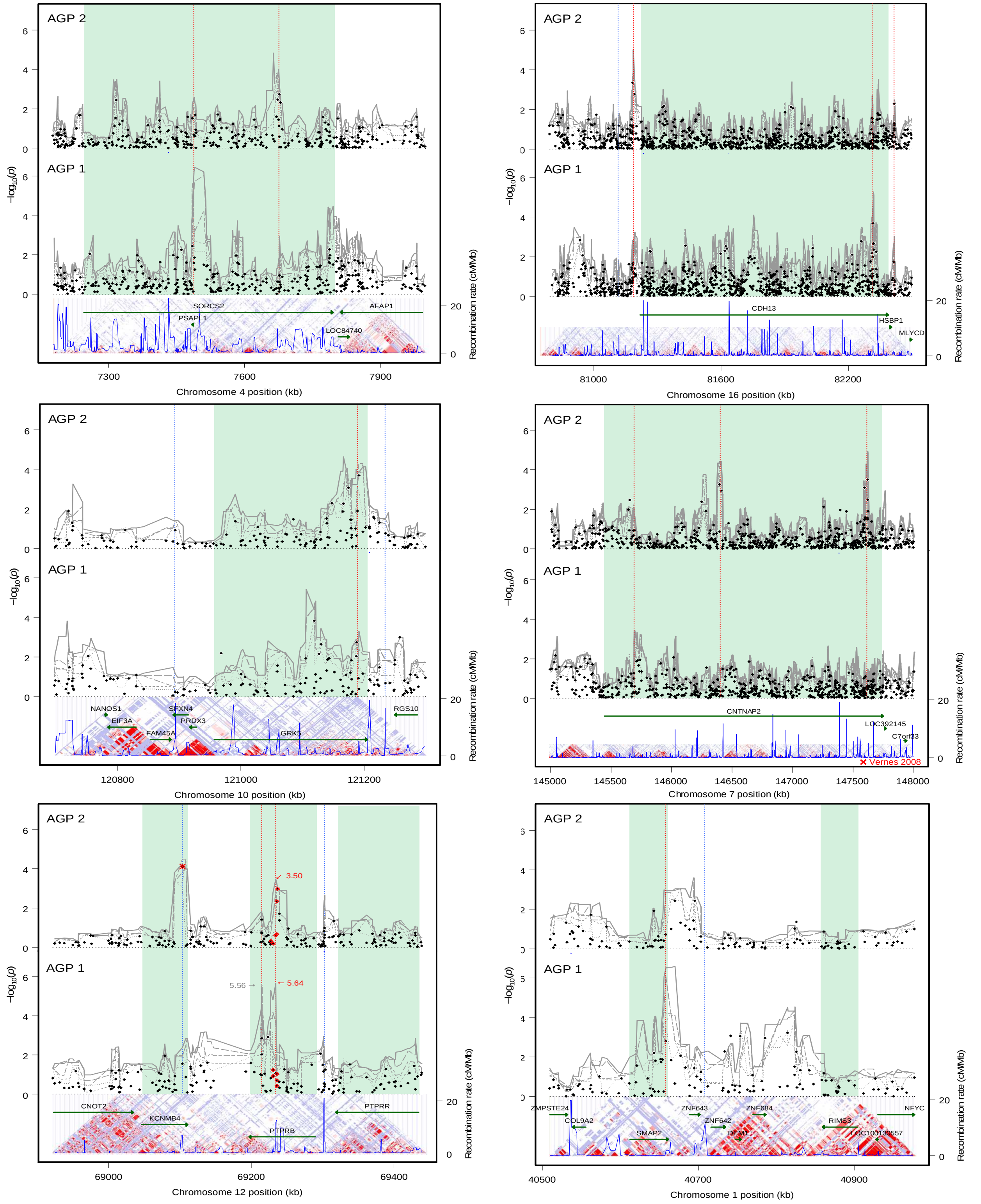

Supplementary Figure 2:. Wide-locus Manhattan plots of selected highly significant regions. The regions most consistently significant in both Stages are *SORCS2* (10th/36th, 11.28), *CDH13* (44th/25th, 10.26), and *GRK5* (35th/59th, 10.06); see Figure 3 for *PTPRT* (90th/20th, 9.95); see Supplementary Figure 1 for *PGR* (65th/18th, 10.17) and *PZP* (33rd/93rd, 9.94). *KCNMB4* exemplifies a region dominated by a single SNP, with two other PTPR, *PTPRB* (21st/880th, 9.11) and *PTPRR*, nearby. See the text for a discussion of *RIMS3* vs, *SMAP2* (8th in AGP I, 6.59) and CNTNAP2 (28th in AGP II, 4.93). Legend: see Figure 3 for details.

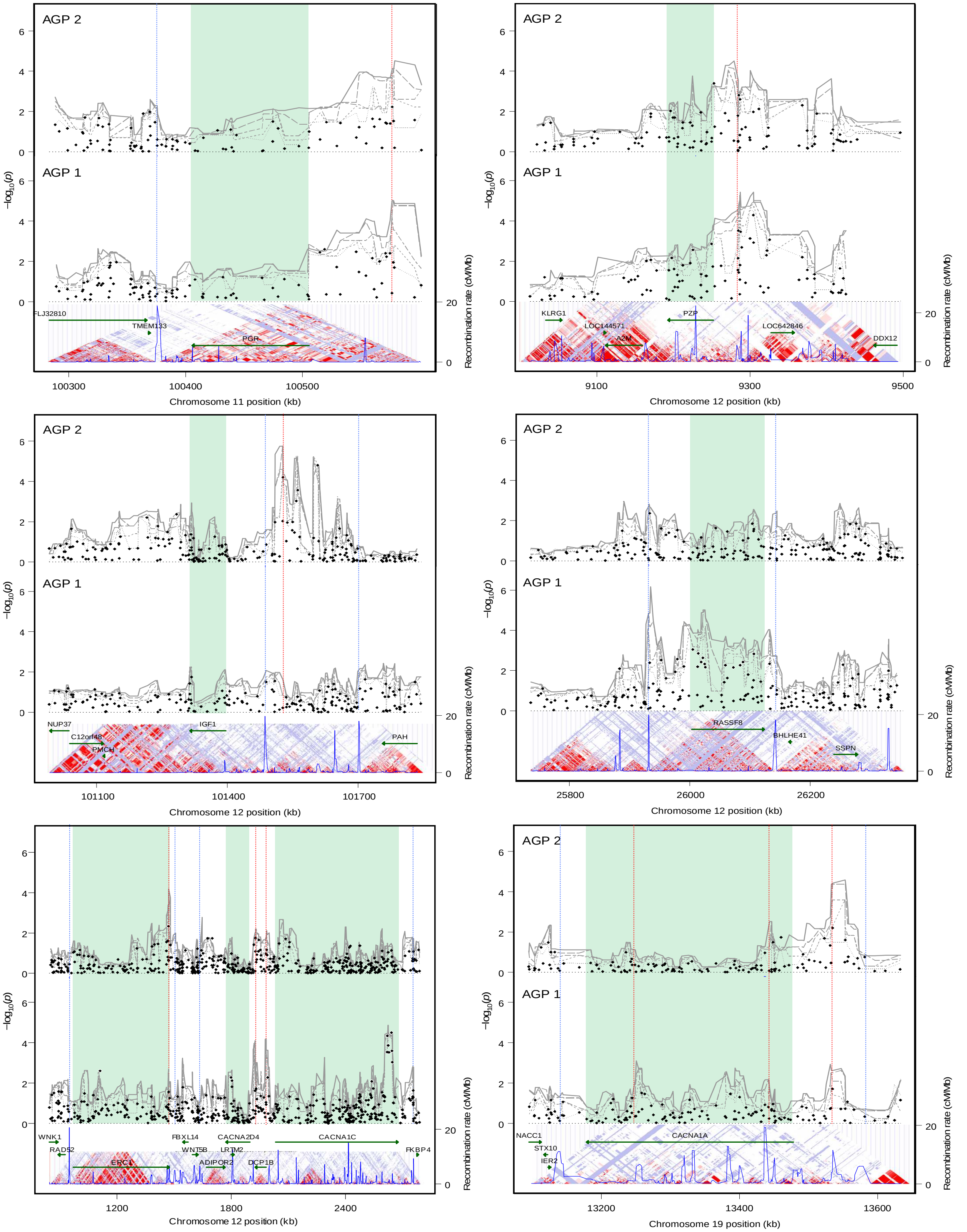

Supplementary Figure 3: Wide-locus Manhattan plots of regions with variations in the promoter region. *PGR*, *PZP*, *IGF1*, *RASSF8*, *CACNA2D4/CACNA1C*, *CACNA1A*; Legend: see Figure 3 for details.

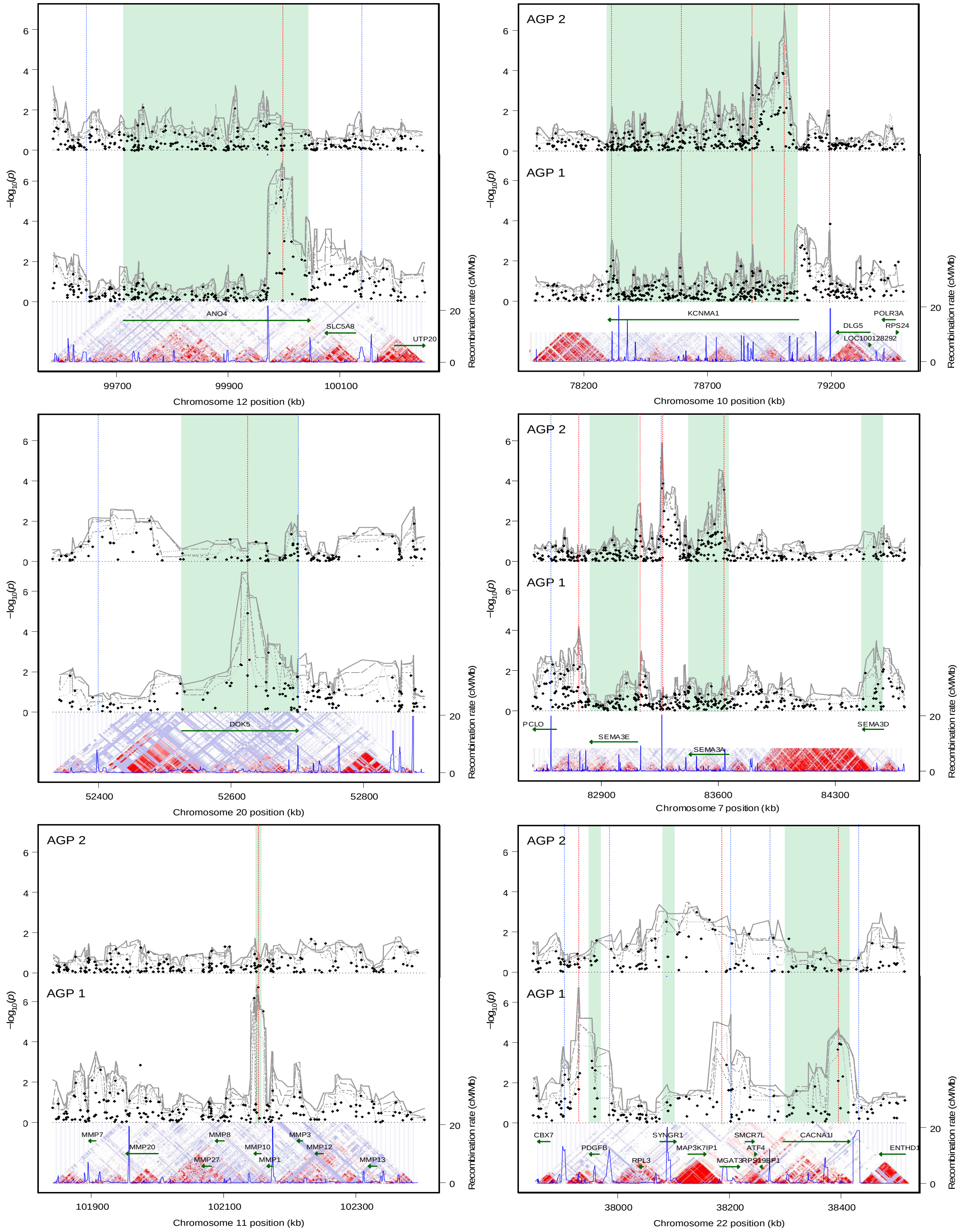

Supplementary Figure 4: Wide-locus Manhattan plots of most significant regions in AGP I (*ANO4*, *DOK5*, *MMP10*, *PDGFB*) and AGP II (*KCNMA1, SEMA3A/SEMA3E*) Legend: see Figure 3 for details.

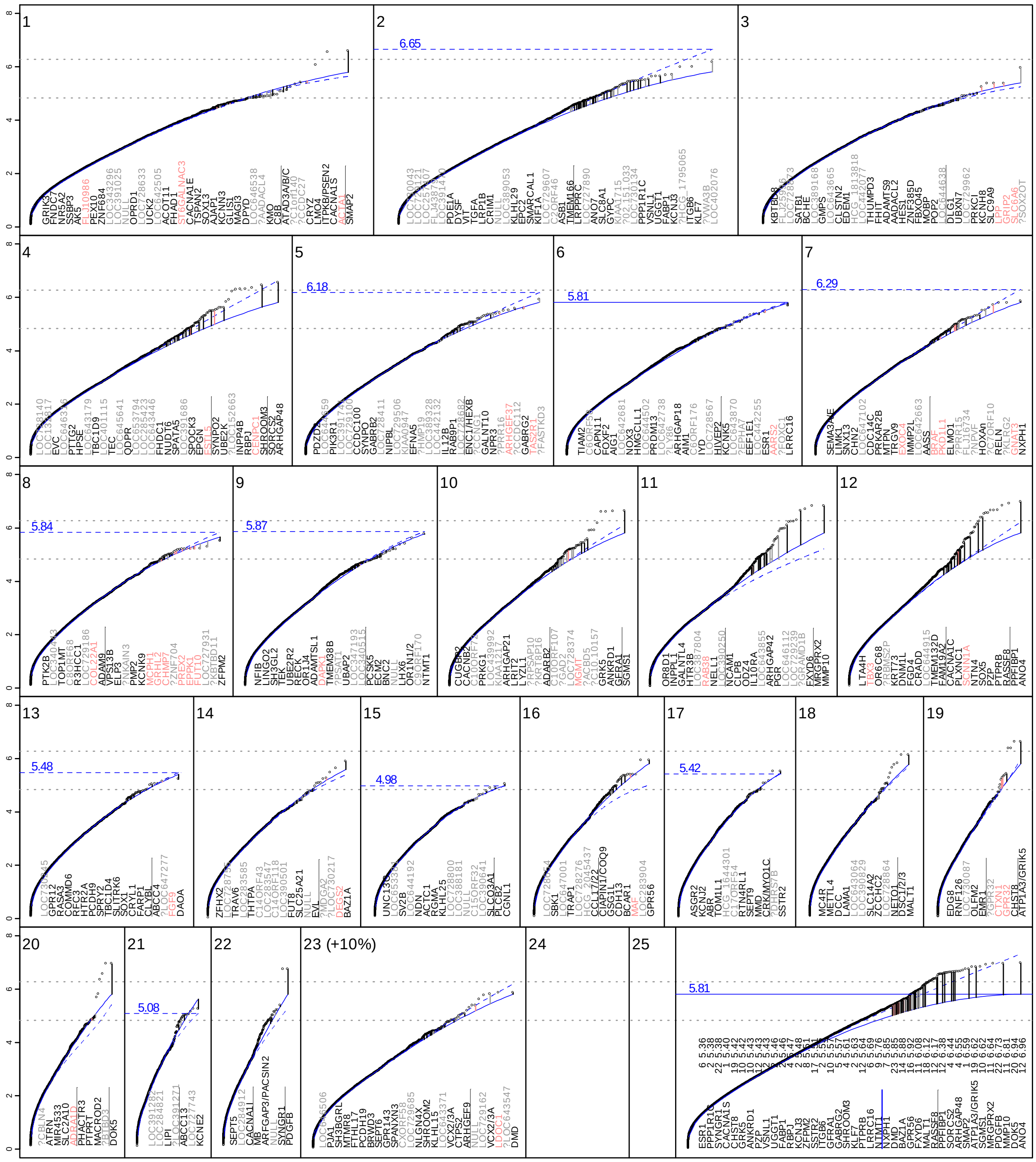

Supplementary Figure 5: µGWAS quantile-rank (QR) plot, AGP I. Each dot represents the most significant result among all diplotypes centered at the same SNP ranked by significance (low to high). Dashed blue curves: loess projection under the null hypothesis. Dashed blue lines: upper limit among the ten chromosomes with the best fit of the projection to the observed s-values. Solid blue line: median cut-off selected among the dashed lines. Solid blue curves: loess projection fitted to the endpoint of the median of the above ten upper limits (see Methods for details, here: chromosome 6, as indicated in the WG plot). ... (continued on Supplementary Figure 4**)**

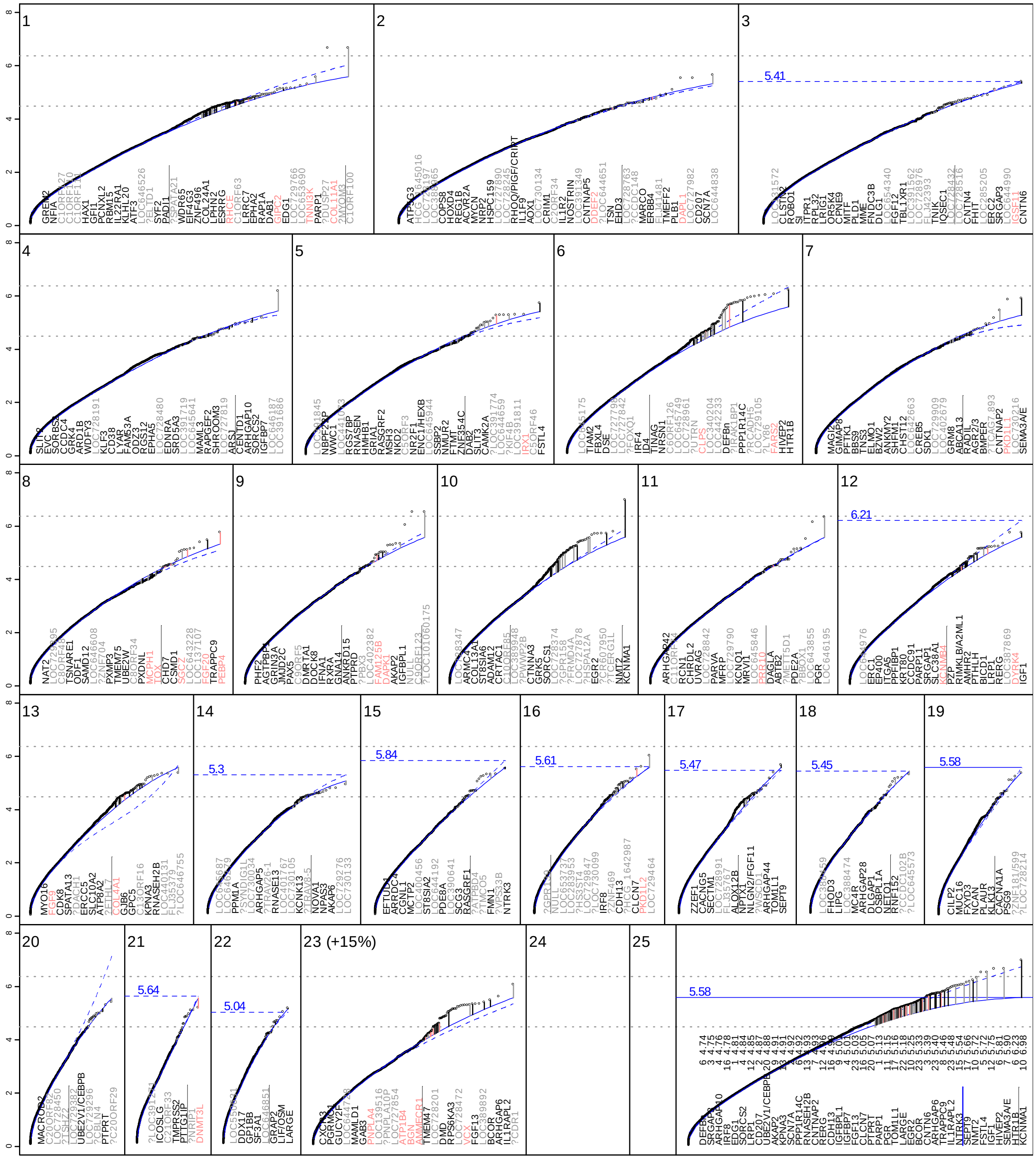

Supplementary Figure 6: µGWAS quantile-rank (QR) plot, AGP II (SDA vs. HFA). (continued from Supplementary Figure 5) ... Vertical lines connect the most significant *s*-values ($-\log_{10} p$) of a gene (dot) with its expected value (solid blue line). Individual chromosomes: Light gray and red names and corresponding vertical lines indicate genes with unknown function and results with low reliability (either low µIC or reliance on a single SNP), respectively. Gene names preceded by "?" refer to regions in distant LD blocks. Whole genome gene list (by significance, right to left) includes chromosome number and s-value; excludes genes with unknown function or low reliability.

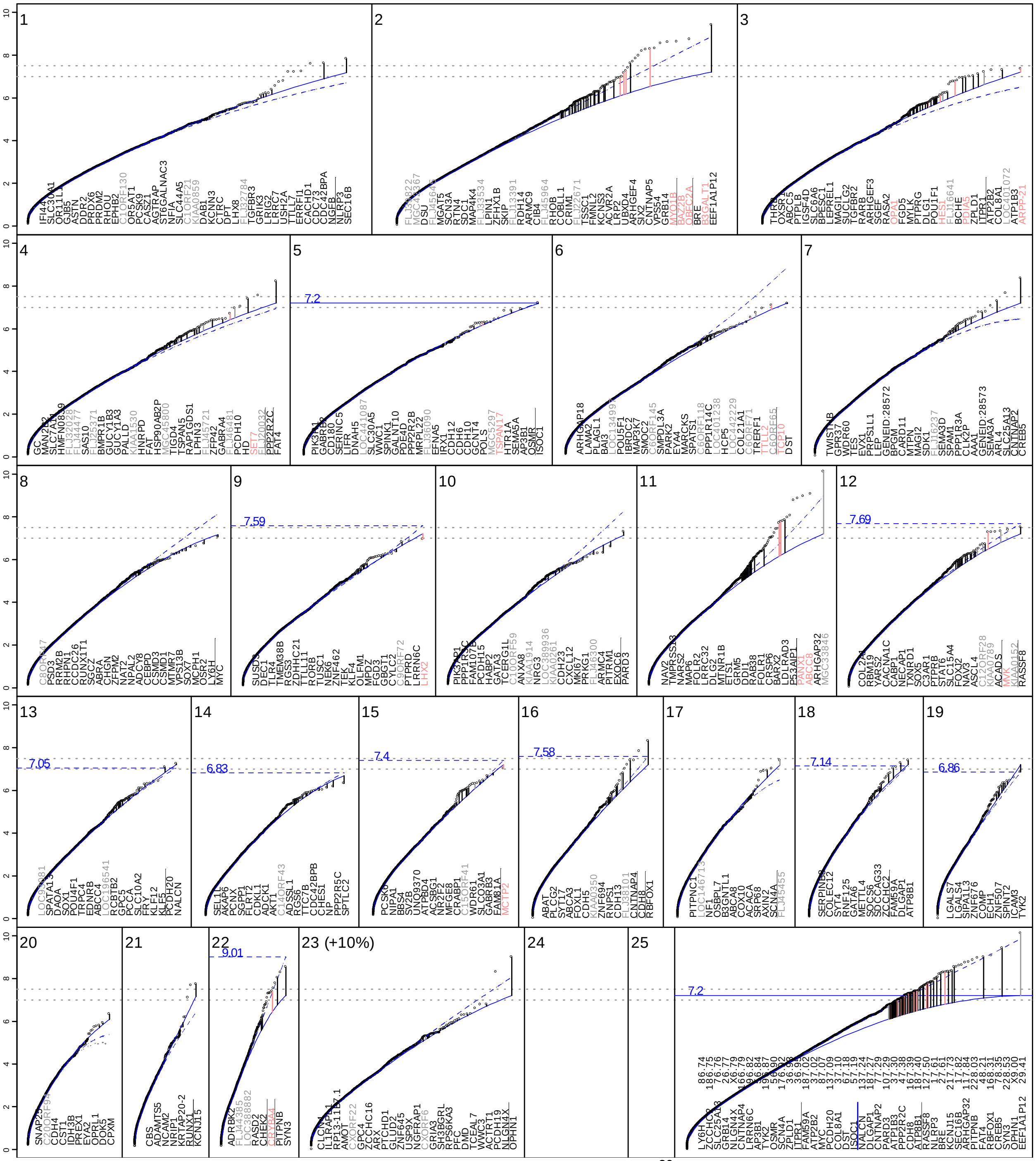

Supplementary Figure 7: µGWAS quantile-rank (QR) plot, CAE[20]. (see Supplementary Figure 5 and Supplementary Figure 6 for legend)

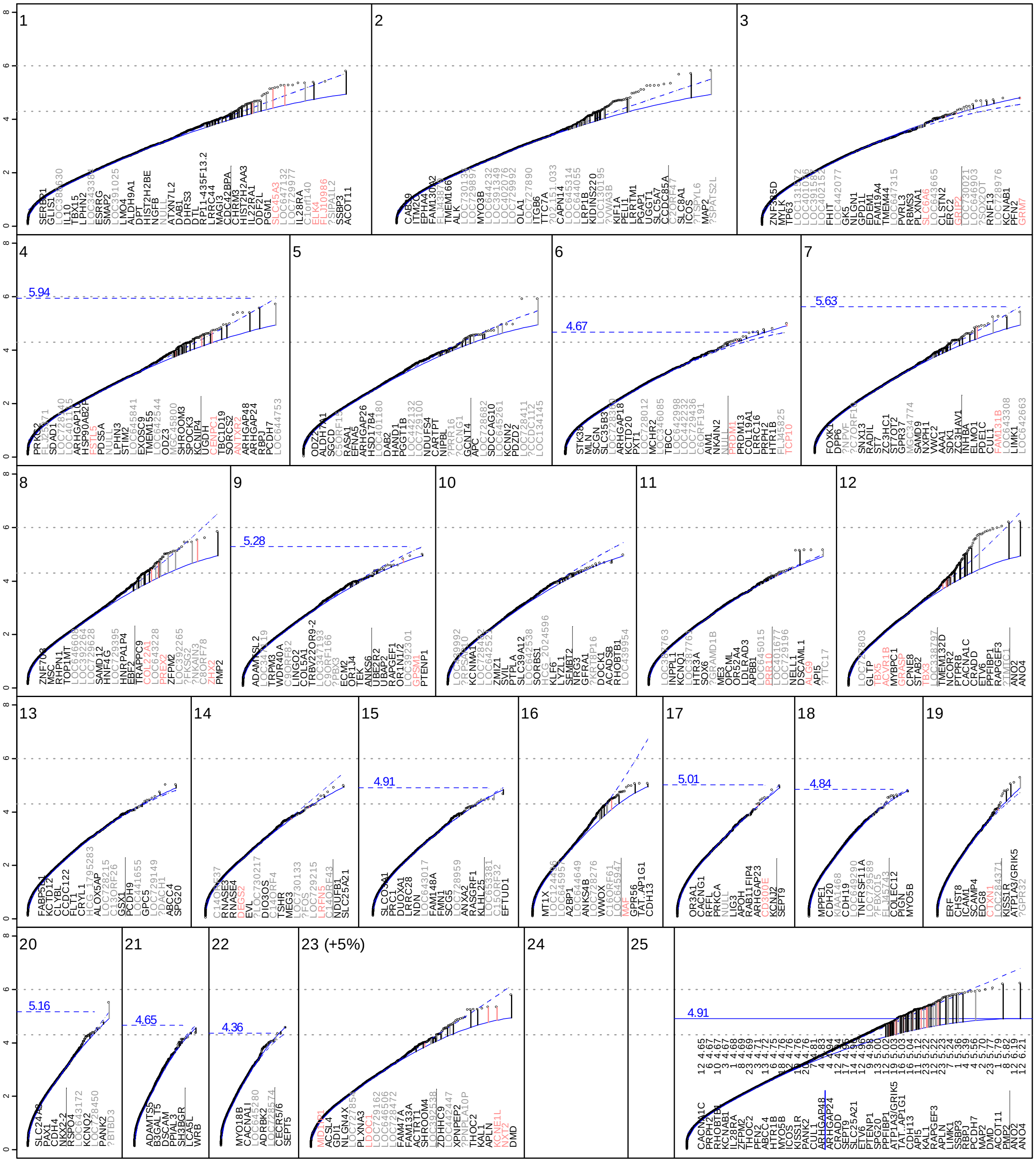

Supplementary Figure 8: µGWAS quantile-rank (QR) plot, AGP I (HFA vs. all parental controls). (see Supplementary Figure 5 and Supplementary Figure 6 for legend)

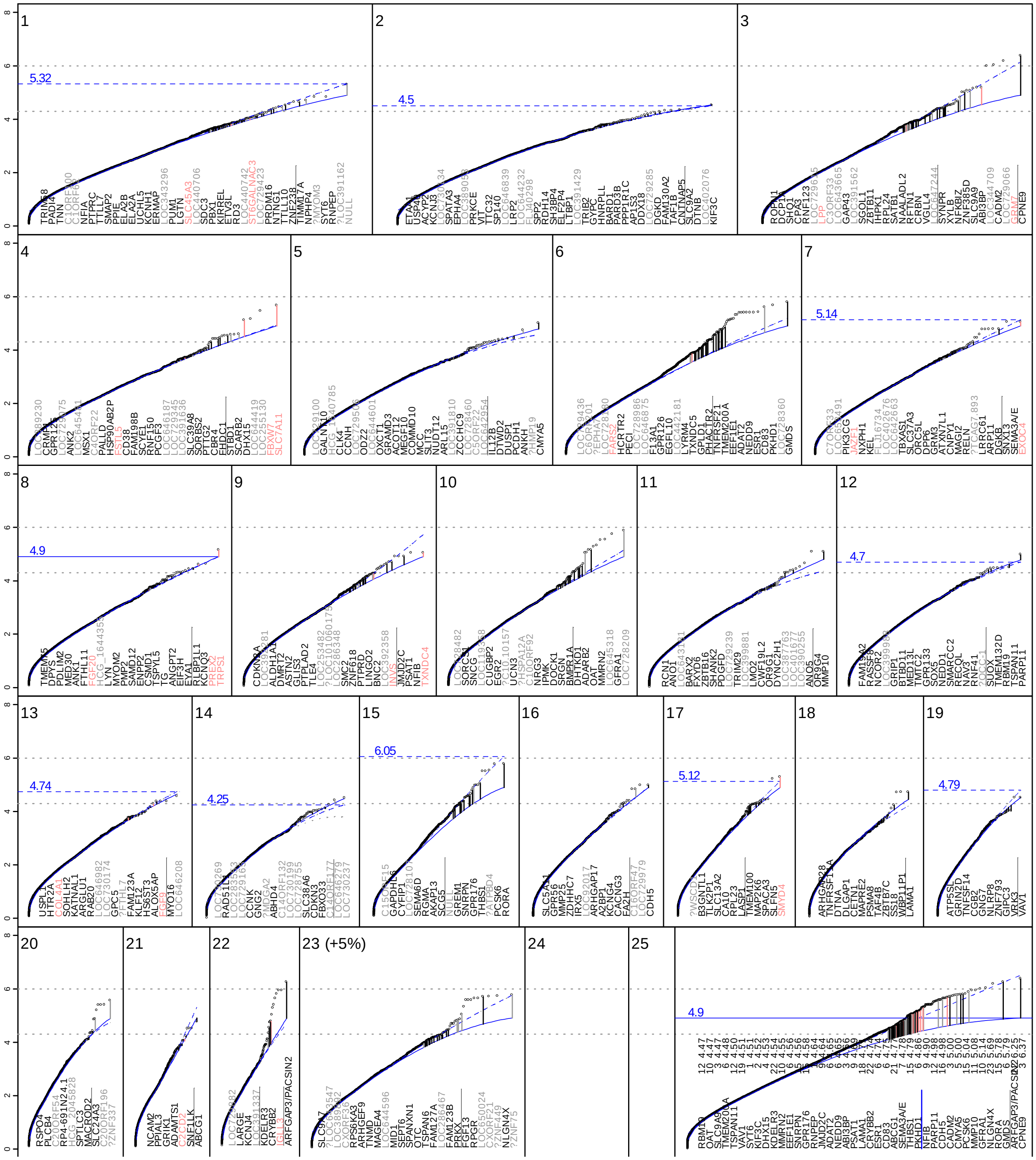

Supplementary Figure 9: µGWAS quantile-rank (QR) plot, AGP I (SDA vs. all parental controls). (see Supplementary Figure 5 and Supplementary Figure 6 for legend)

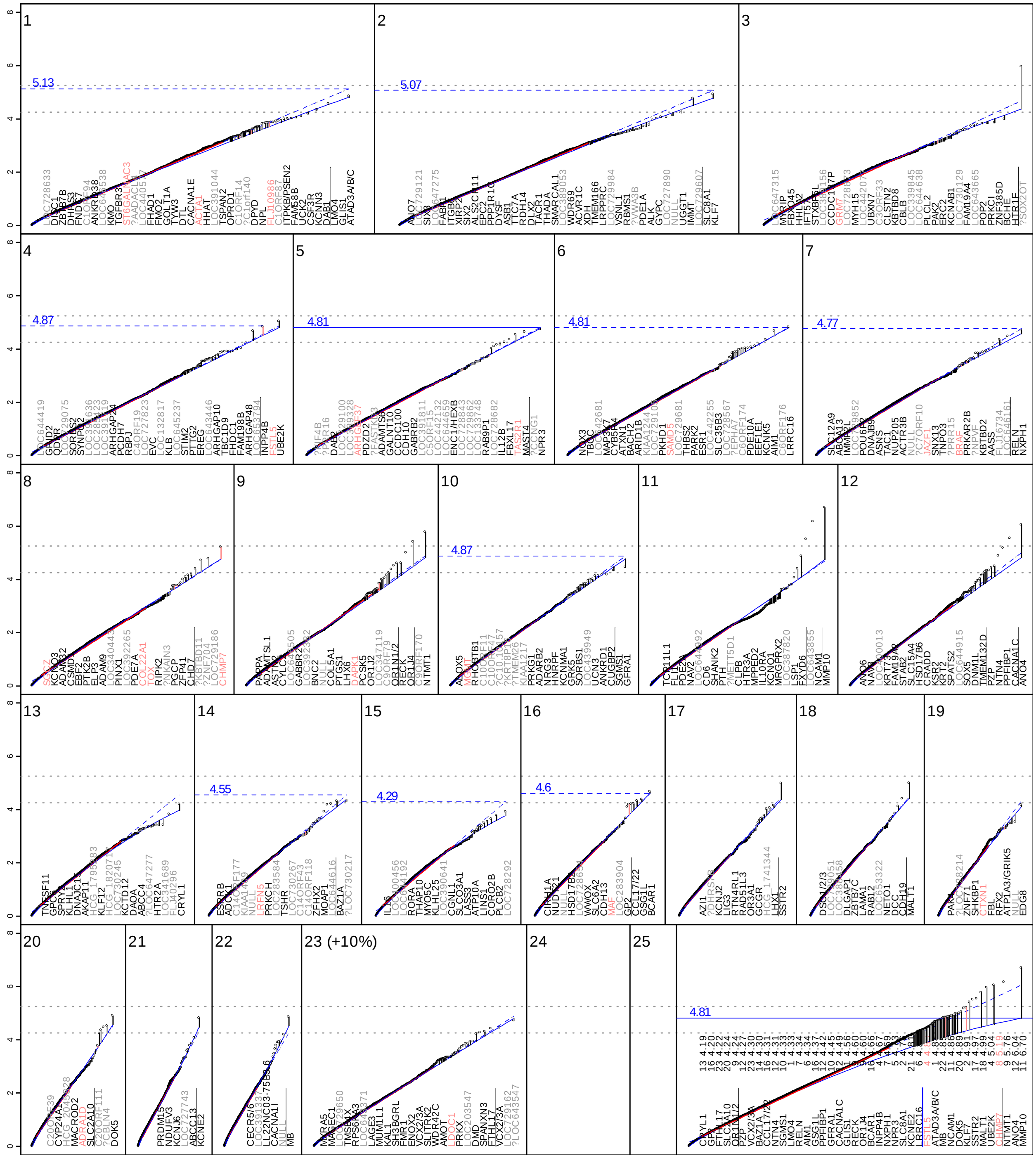

Supplementary Figure 10: ssGWAS quantile-rank (QR) plot, AGP I. (see Supplementary Figure 5 and Supplementary Figure 6 for legend)

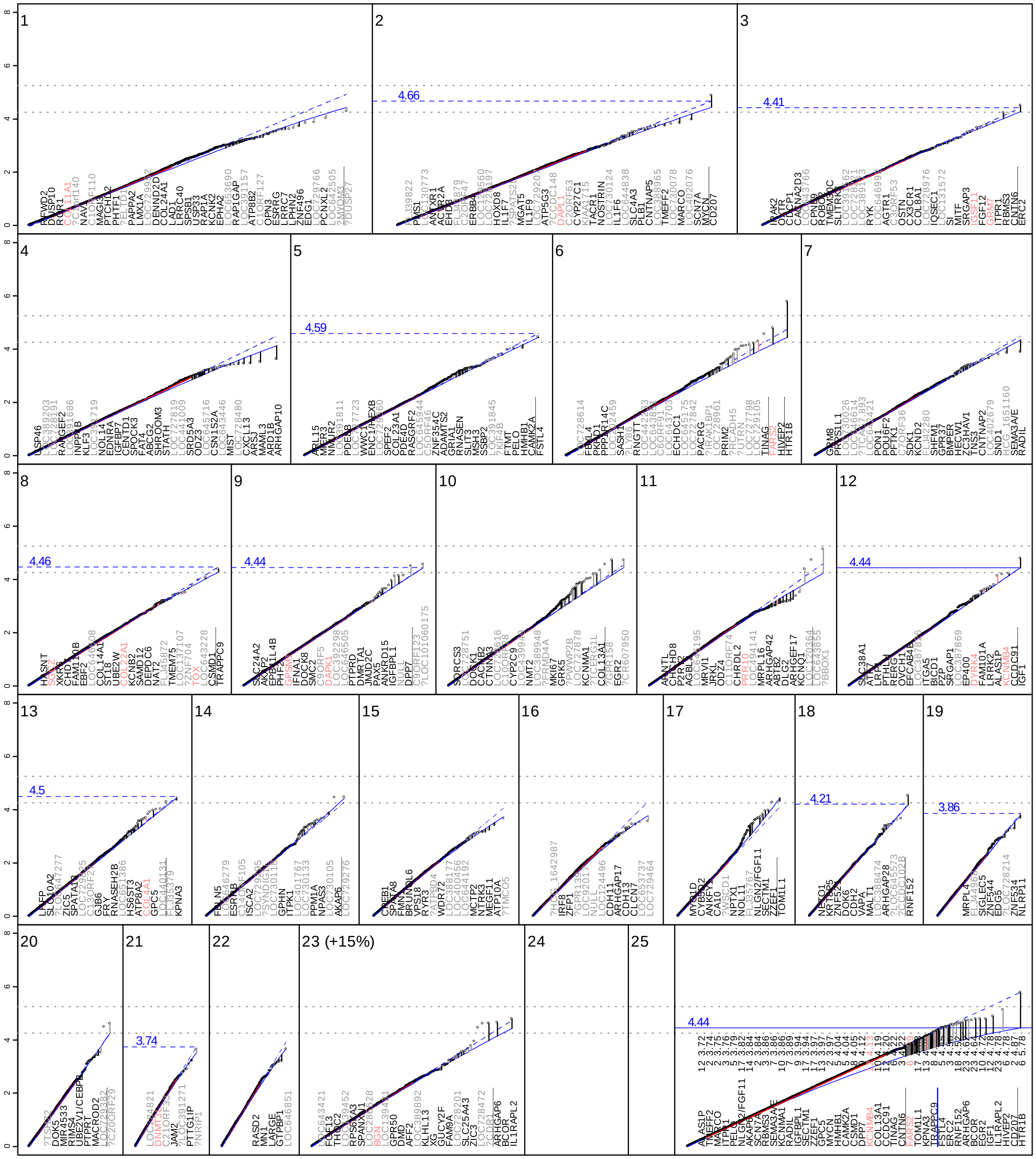

Supplementary Figure 11: ssGWAS quantile-rank (QR) plot, AGP II. (see Supplementary Figure 5 and Supplementary Figure 6 for legend)

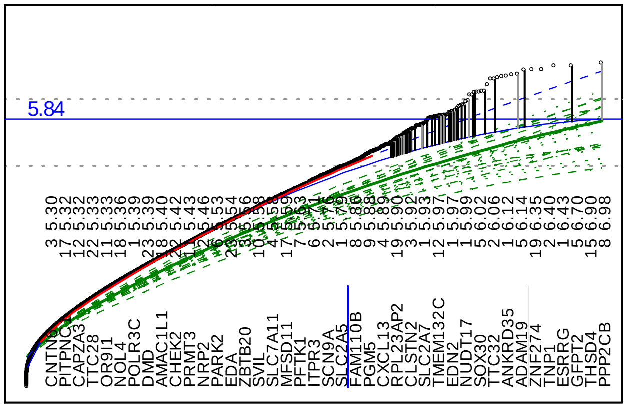

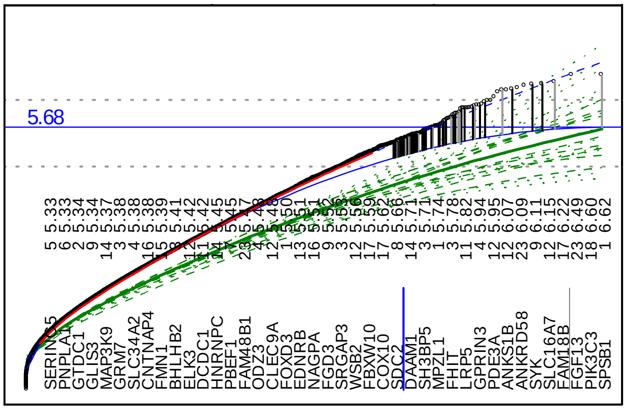

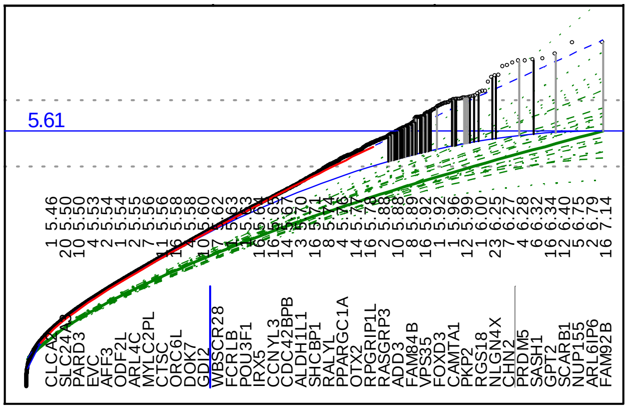

Supplementary Figure 12: randomization vs. low variance selection.

Left: results of three WG analyses using randomly permuted phenotypes. Dashed green lines show the distributions of the fits from the ten chromosomes with the lowest variance around the loess fit. Dotted green lines show the distributions of the fits from the remaining 12 chromosomes (chromosome 23 is excluded from these calculations). The solid green line indicates one of the distributions whose endpoint is closest to the median of the above ten 'good fit' distributions, which, of course, can differ across simulations. Genes known to have no known function are excluded from the list shown.

Overall, up to 10% of all genes with known function can be associated with Ras/Ca^{2+} signaling. Moreover, association with disease severity typically increases MAF and, thus, the chance for Ras/Ca^{2+} related genes to be among the most significant for any permutation of the phenotypes (see Methods). Hence, about ten related genes among the top 41 shown (20 percent enrichment) are to be expected under the permutations, compared to 34 or 24 related genes (82 or 58 percent enrichment for the original phenotypes in AGP I or II, respectively, see Supplementary Table 1).

Bottom left: For each chromosome, the median of the three randomizations shown above is selected.

Compared to a median of only six genes reaching the empirical 6.30 level of significance with permuted phenotypes, eleven genes are above this cut-off with the original phenotype.

Bottom right: For direct comparison, the estimate based on the actual data using the chromosomes with good fit, as shown in Supplementary Figure 4.

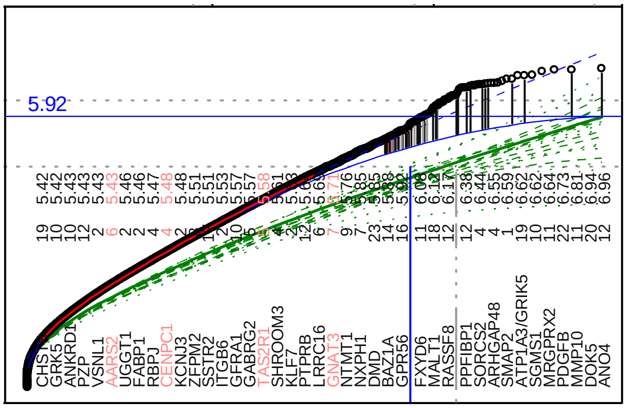

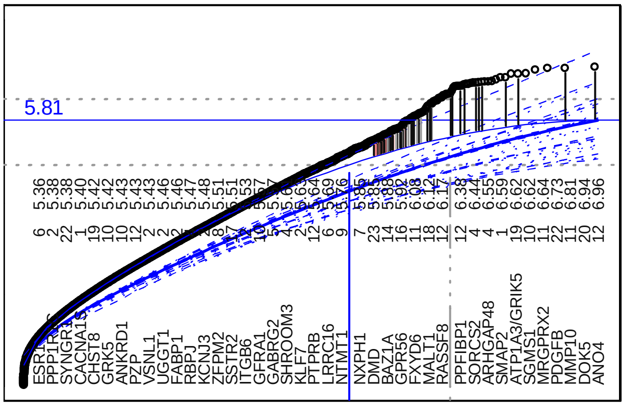

Supplementary Table 1: Top 100 regions by µGWAS in AGP I (left) and AGP II (right) and most closely related genes in SFARI Gene. s: $-\log_{10}(p)$, s_F: Fisher($s_{AGP\ I}$, $s_{AGP\ II}$). Genes highlighted through color coding are included in Figure 2, top and center, for AGP I and AGP II, respectively.

Seq	Chr	Coordinate	Gene	Entrez	s	55	SFARI	sF	Match	Match	sF	Chr	Coordinate	Gene	Entrez	s	46	SFARI
1	12	99,997,403	ANO4	121601	6.964	1				795	**9.38**	10	79,013,206	KCNMA1	3778	6.979	1	KCNMA1
2	20	52,616,118	DOK5	55816	6.940	1		**9.29**	497			6	77,822,690	HTR1B	3351	6.227		HTR1B
3	11	102,149,811	MMP10	4319	6.812	1				388	**8.72**	7	83,262,044	SEMA3A/E		5.901	1	SEMA5A
4	22	37,929,247	PDGFB	5155	6.733	1				110	**9.22**	6	143,054,474	HIVEP2	3097	5.811		
5	11	19,034,480	MRGPRX2	117194	6.636	1						12	101,519,877	IGF1	3479	5.747	1	
6	10	51,827,941	SGMS1	259230	6.625	1	SGSM3					5	132,800,280	FSTL4	23105	5.719		
7	19	47,234,675	ATP1A3/GRIK5		6.621	1	GRIK2					10	15,240,566	NMT2	9397	5.717		
8	1	40,670,079	SMAP2	64744	6.587	1	RIMS3			123	**9.01**	17	73,082,372	SEPT9	10801	5.665	1	
9	4	90,290,720	ARHGAP48	10144	6.546	1	ARHGAPn					15	86,391,117	NTRK3	4916	5.536	1	NTRK3
10	4	7,490,276	SORCS2	57537	6.443	1		**9.85**	36			23	104,381,002	IL1RAPL2	26280	5.477	1	IL1RAPL2
11	12	27,522,647	PPFIBP1	5792	6.376	1	PTPRx	**9.17**	197	633	8.04	8	141,190,927	TRAPPC9	83696	5.464	1	
12	12	25,934,704	RASSF8	11228	6.168	1	RASSF5					23	11,618,552	ARHGAP6	395	5.400	1	ARHGAPn
13	18	54,617,513	MALT1	10892	6.118			8.24	841			3	1,297,885	CNTN6	27255	5.391	1	CNTN6
14	11	117,236,346	FXYD6	53826	6.084	1						23	39,778,167	BCOR	54880	5.327		
15	16	56,212,982	GPR56	9289	5.924	1		8.20	620			10	64,115,570	EGR2	1959	5.247	1	EGR2
16	14	34,407,116	BAZ1A	11177	5.878	1				440	7.97	22	32,339,001	LARGE	9215	5.183		
17	23	31,989,875	DMD	1756	5.852	1	DMD	**8.97**	88	186	8.36	17	50,320,792	TOM1L1	10040	5.158		
18	7	9,128,496	NXPH1	30010	5.846	1	SG			66	**8.78**	11	100,603,405	PGR	5241	5.149	1	
19	9	131,397,657	NTMT1	28989	5.765							1	224,760,165	PARP1	142	5.129		
20	6	25,533,505	LRRC16A	55604	5.689					90	**8.57**	20	40,415,101	PTPRT	11122	5.066	1	PTPRT
21	12	69,234,337	PTPRB	5787	5.644	1	PTPRx	7.77	880	503	7.77	16	1,428,647	CLCN7	1186	5.048	1	
22	2	207,729,186	KLF7	8609	5.634	1						23	137,452,143	FGF13	2258	5.034		FGFBP3
23	4	77,587,494	SHROOM3	57619	5.607	1		**8.66**	109			4	57,601,443	IGFBP7	3490	5.009	1	
24	5	161,378,809	GABRG2	2566	5.574	1	GABRxn					9	38,399,081	IGFBPL1	347252	5.007	1	
25	10	117,851,287	GFRA1	2674	5.568	1				45	**8.86**	16	81,186,530	CDH13	1012	4.989	1	CDHn
26	2	160,611,093	ITGB6	3694	5.530	1						12	15,210,855	RERG	85004	4.963		
27	17	68,662,948	SSTR2	6752	5.511					626	7.54	13	50,372,054	RNASEH2B	79621	4.933		
28	8	106,945,510	ZFPM2	23414	5.509							7	147,621,670	CNTNAP2	26047	4.933	1	CNTNAP2
29	2	155,276,308	KCNJ3	3760	5.485	1	KCNJ10					6	150,653,078	PPP1R14C	81706	4.917	1	PPP1Rnx
30	4	26,069,146	RBPJ	3516	5.473	1						2	166,951,683	SCN7A	6332	4.915	1	SCNnA
31	2	88,209,949	FABP1	2168	5.464		FABP7					13	49,154,441	KPNA3	3839	4.909		
32	2	128,649,230	UGGT1	56886	5.455	1				956	7.31	9	111,968,951	AKAP2	11217	4.908	1	
33	2	17,261,376	VSNL1	7447	5.433	1						20	48,236,776	UBE2V1/CEBPB		4.880		
34	12	9,304,709	PZP	5858	5.432	1		**8.56**	93			2	70,913,619	CD207	50489	4.873		
35	10	92,773,493	ANKRD1	27063	5.427		ANKRD11					12	55,817,899	LRP1	4035	4.851	1	
36	10	121,106,202	GRK5	2869	5.424	1		**8.68**	59	10	**9.85**	4	7,663,672	SORCS2	57537	4.836	1	
37	19	38,835,881	CHST8	64377	5.415		CHST11					1	101,517,409	EDG1	1901	4.813		
38	1	199,196,628	CACNA1S	779	5.403	1	CACNA1x					16	84,506,772	IRF8	3394	4.775		
39	22	38,202,646	SYNGR1	9145	5.385	1		7.53	871	636	7.37	4	149,401,007	ARHGAP10	79658	4.762	1	ARHGAPnx
40	2	182,684,972	PPP1R1C	151242	5.377	1	PPP1Rnx					3	9,225,949	SRGAP3	9901	4.752	1	
41	6	152,134,442	ESR1	2099	5.359	1	ESR1					6	50,119,755	DEFB110	245913	4.745		
42	16	73,793,655	BCAR1	9564	5.348	1						11	72,008,094	PDE2A	5138	4.733		PDEnx
43	5	32,781,509	NPR3	4883	5.346							13	90,936,596	GPC5	2262	4.712		GPCn
44	7	103,231,266	RELN	5649	5.334	1	RELN					2	28,653,279	PLB1	151056	4.707		
45	16	82,318,009	CDH13	1012	5.266	1		**8.86**	25	399	7.56	1	57,415,859	DAB1	1600	4.704	1	DAB1
46	21	34,514,668	KCNE2	9992	5.252	1						15	30,874,881	FMN1	342184	4.700		
47	4	143,143,541	INPP4B	8821	5.252	1	INPP1					10	108,270,642	SORCS1	114815	4.686	1	
48	13	104,999,215	DAOA	267012	5.221	1						1	112,049,488	RAP1A	5906	4.681	1	
49	9	124,344,571	OR1N1/2		5.184							3	55,645,960	ERC2	26059	4.680	1	
50	7	27,168,814	HOXA1	3198	5.162		HOXA1					19	48,464,142	PSG9	5678	4.674		
51	12	24,436,518	SOX5	6660	5.158							1	16,294,597	EPHA2	1969	4.673		
52	12	94,801,931	NTN4	59277	5.145	1		7.39	698			1	69,932,766	LRRC7	57554	4.673	1	
53	1	224,967,038	ITPKB/PSEN2		5.113	1						11	34,268,213	ABTB2	25841	4.664		
54	2	127,232,976	GYPC	2995	5.077							22	28,968,226	LIF/OSM		4.659		
55	23	8,286,488	VCX2/3A		5.077			7.33	691			5	149,613,148	CAMK2A	815	4.651	1	
56	4	39,378,484	UBE2K	3093	5.074							1	214,906,994	ESRRG	2104	4.651	1	
57	6	8,016,561	EEF1E1	9521	5.072					433	7.46	1	82,331,131	LPHN2	23266	4.641		
58	18	26,927,892	DSC1/2/3		5.063			7.52	452			12	32,394,809	BICD1	636	4.640		
59	9	124,019,951	LHX6	26468	5.061					36	**8.68**	10	121,166,591	GRK5	2869	4.639	1	
60	5	153,782,557	GALNT10	55568	5.061							12	28,074,553	PTHLH	5744	4.623		
61	1	87,518,905	LMO4	8543	5.059			7.36	634			4	35,682,788	CENTD1	116984	4.618		
62	12	93,321,870	PLXNC1	10154	5.041	1		7.30	690			13	19,745,138	GJB6	10804	4.616		
63	15	55,638,868	CGNL1	84952	5.037			7.70	305			2	192,529,143	TMEFF2	23671	4.615		
64	2	40,527,812	SLC8A1	6546	5.036	1						17	12,793,117	ARHGAP44	9912	4.613	1	
65	13	94,633,879	ABCC4	10257	5.027							10	67,799,789	CTNNA3	29119	4.608		
66	11	100,576,775	PGR	5241	5.023	1		**8.78**	18			14	31,921,296	AKAP6	9472	4.603	1	
67	8	140,422,530	KCNK9	51305	5.022	1				631	7.23	14	32,511,542	NPAS3	64067	4.602		
68	21	14,650,668	ABCC13	150000	5.018							12	52,121,210	AMHR2	269	4.599		
69	11	100,237,862	ARHGAP42	143872	5.006	1		7.75	249			12	8,841,975	RIMKLB/A2ML1		4.592	1	
70	11	117,335,639	IL10RA	3587	4.992					210	7.77	3	60,481,389	FHIT	2272	4.592		
71	2	241,715,503	ANO7	50636	4.992	1				727	7.15	2	212,873,037	ERBB4	2066	4.592	1	
72	3	144,628,258	SLC9A9	285195	4.980	1						17	7,344,666	NLGN2/FGF11		4.589	1	
73	7	36,936,699	ELMO1	9844	4.970			7.69	262			19	13,552,951	CACNA1A	773	4.586	1	
74	16	27,875,165	GSG1L	146395	4.956							17	76,055,642	NPTX1	4884	4.586		
75	3	19,557,427	KCNH8	131096	4.954	1						5	168,682,315	SLIT3	6586	4.586		
76	3	171,304,895	PRKCI	5584	4.949	1						1	86,185,610	COL24A1	255631	4.582		
77	1	74,930,706	CRYZ	1429	4.923							1	245,526,979	ZNF496	84838	4.578		
78	22	41,553,146	ARFGAP3	26286	4.922	1				837	7.06	3	2,402,611	CNTN4	152330	4.575	1	
79	20	15,095,150	MACROD2	140733	4.913			7.85	155			23	20,492,176	RPS6KA3	6197	4.575		
80	9	16,618,828	BNC2	54796	4.913			7.13	766			2	119,441,621	MARCO	8685	4.572		
81	8	28,059,736	ELP3	55140	4.913							1	20,987,420	EIF4G3	8672	4.571		
82	11	78,711,687	ODZ4	26011	4.910							1	43,414,654	WDR65	149465	4.553		
83	5	73,996,748	ENC1/HEXB		4.907			7.77	190	694	7.13	7	33,869,108	BMPER	168667	4.546		
84	8	100,867,916	VPS13B	157680	4.903					519	7.26	9	10,020,458	PTPRD	5789	4.530	1	
85	3	197,642,729	UBXN7	26043	4.902							18	57,595,661	RNF152	220441	4.524		
86	11	71,862,239	CLPB	81570	4.893							6	24,241,451	NRSN1	140767	4.523		
87	3	198,234,604	MFI2	4241	4.892					218	7.67	8	3,490,417	CSMD1	64478	4.521		
88	9	94,314,082	ECM2	1842	4.890					17	**8.97**	23	31,119,190	DMD	1756	4.517	1	
89	15	38,404,501	PLCB2	5330	4.889	1						14	25,633,636	NOVA1	4857	4.517		
90	20	40,415,519	PTPRT	11122	4.887	1		**8.57**	20			7	16,887,386	AGR2/3		4.516		
91	12	2,624,388	CACNA1C	775	4.870	1						6	54,307,077	TINAG	27283	4.516		
92	16	56,040,691	CIAPIN1/COQ9		4.860							11	61,237,424	DAGLA	747	4.513	1	
93	9	77,952,616	PCSK5	5125	4.858					34	**8.56**	12	9,279,277	PZP	5858	4.510	1	
94	11	112,608,469	NCAM1	4684	4.856	1		7.04	844			7	4,783,100	RADIL	55698	4.507	1	
95	22	34,337,021	MB	4151	4.853					161	7.81	8	61,960,444	CHD7	55636	4.501		
96	20	57,589,240	PHACTR3	116154	4.851							9	649,124	ANKRD15	23189	4.497		
97	2	44,091,378	LRPPRC	10128	4.849							5	39,475,063	DAB2	1601	4.491	1	
98	4	120,237,615	SYNPO2	171024	4.848							22	38,668,230	GRAP2	9402	4.487	1	
99	1	1,394,672	ATAD3A/B/C		4.847							23	34,342,354	TMEM47	83604	4.486		
100	6	39,191,266	KCNK5	8645	4.839	1				999	6.88	4	115,229,130	ARSJ		4.479	1	

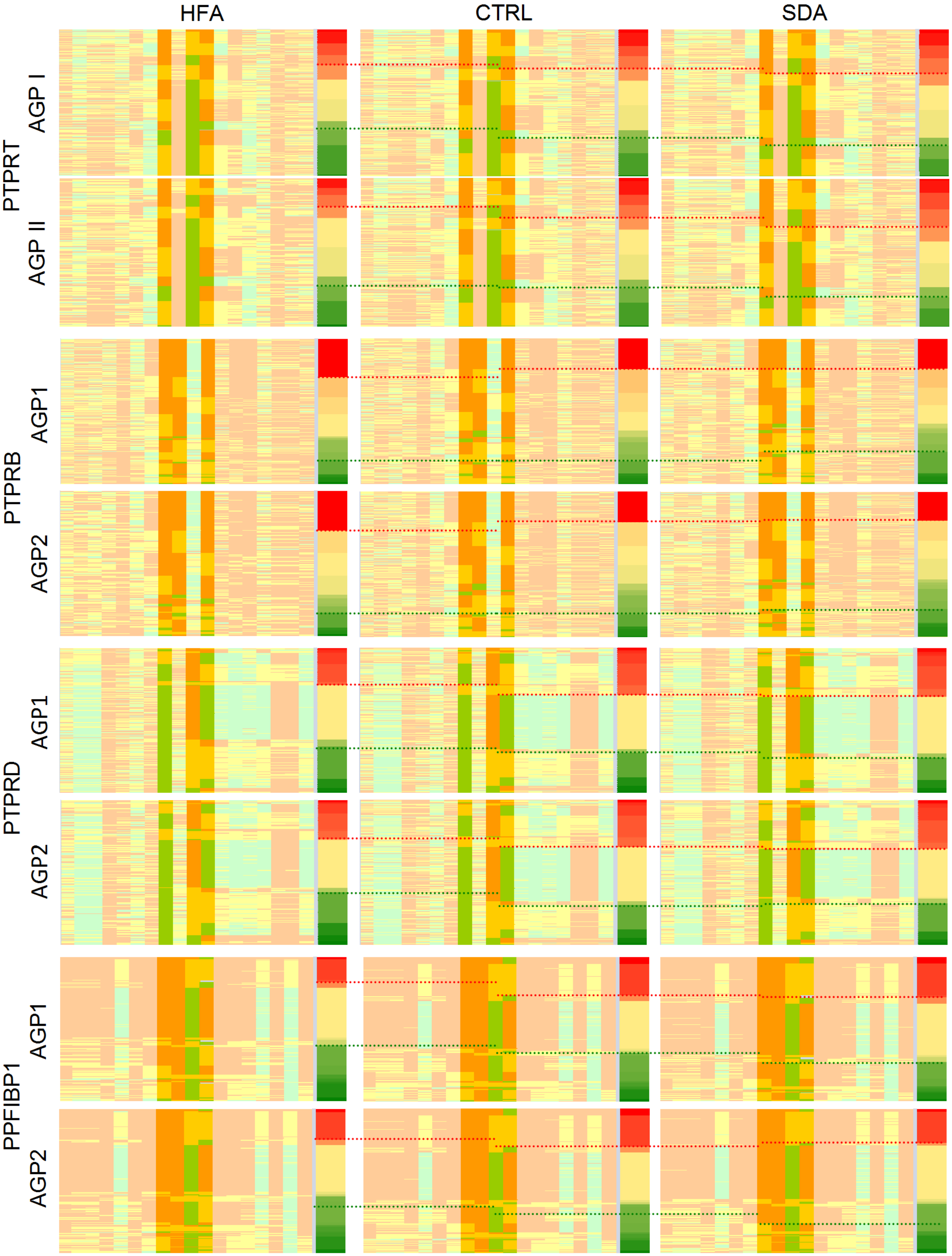
HFA
CTRL
SDA
PTPRT
AGP I
AGP II
PTPRB
AGP1
AGP2
PTPRD
AGP1
AGP2
PPFIBP1
AGP1
AGP2

Supplementary Figure 13: Comparison of SDA and HFA cases with melanoma controls. *PTPRT*: rs6102794, -rs6072694, and rs6102795; *PTPRB*: rs4761222, rs2303963, and rs2116211; *PTPRD*:-rs4742447, rs7020466, -rs6477258; *PPFIBP1*: rs411432, rs374484, -rs411816, rs6487610. See Figure 4 for legend.

Supplementary Table 2: Enrichment of Most Significant Regions With Functionally Related Genes. Heuristical enrichment was determined based on the top 200 regions (see Supplementary Table 1 for the first 100). (As the structure of the particular network configuration is highly variable, the focus here is on stability of enrichment within a given structure.) Network results (yellow): Networks are based on direct relationships, using human and mouse information with at least high confidence, excluding miRNA data bases. Based on gaps in score/number of focus molecules (highlighted in dark yellow), the top one or two networks are shown. Annotation results (gray): Groups of genes by functional annotation were chosen based on reported p-values a sorting criterion. For regions of size >20, sets of n < 4 molecules were excluded to avoid artifacts, as were sets of n > 60 molecules for lack of specificity. The most consistent gene sets by functional annotation (AGP I: Schizophrenia, AGP II: neuritogenesis) are highlighted as boxed.

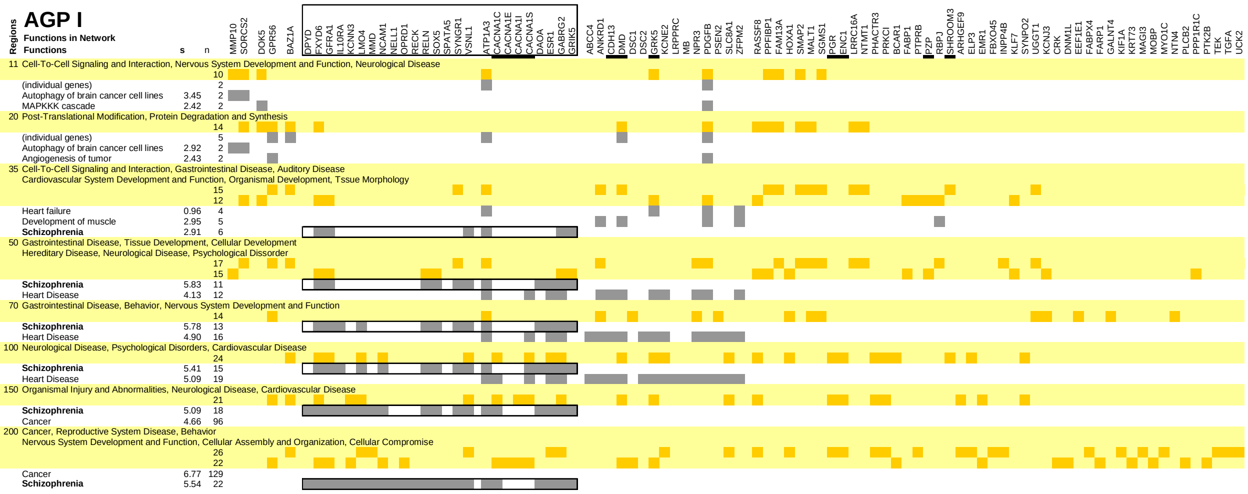

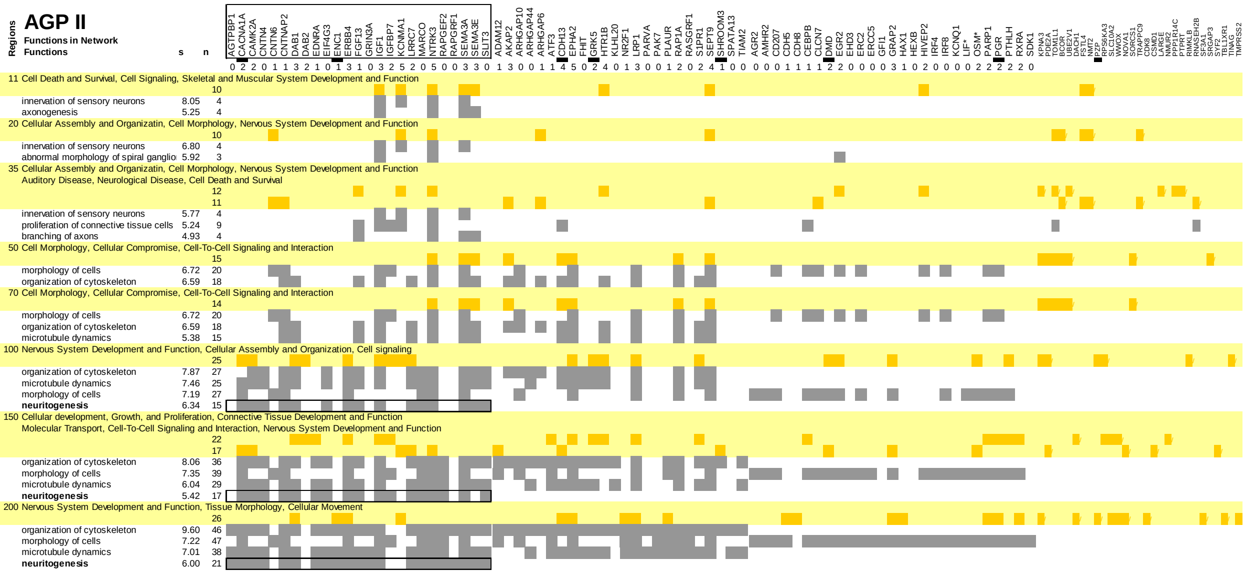

Supplement: Supplementary Information [file tp2013124x1.pdf]
